# Relationship between *EGF*, *TGFA*, and *EGFR* Gene Polymorphisms and Traditional Chinese Medicine ZHENG in Gastric Cancer

**DOI:** 10.1155/2013/731071

**Published:** 2013-12-16

**Authors:** Junfeng Zhang, Zhen Zhan, Juan Wu, Chunbing Zhang, Yaping Yang, Shujuan Tong, Ruiping Wang, Xuewen Yang, Wei Dong, Yajun Chen

**Affiliations:** ^1^Discipline of Chinese and Western Integrative Medicine, Nanjing University of Chinese Medicine, Nanjing 210023, China; ^2^College of Basic Medicine, Nanjing University of Chinese Medicine, Nanjing 210023, China; ^3^Department of Clinical Laboratory, First Affiliated Hospital of Nanjing University of Chinese Medicine, Nanjing 210029, China; ^4^Department of TCM Oncology, First Affiliated Hospital of Nanjing University of Chinese Medicine, Nanjing 210029, China

## Abstract

In traditional Chinese medicine (TCM), correct syndrome differentiation is the most important principle guiding the prescription of Chinese herbal formulae for the treatment of gastric cancer (GC). We aimed to reveal the genetic mechanisms underlying GC syndrome differentiation (ZHENG) in a population of 387 GC patients. Twenty-nine single nucleotide polymorphisms (SNPs) in *EGF*, *TGFA*, and *EGFR* were investigated. Two SNPs, rs11466285 in *TGFA* and rs884225 in *EGFR*, were significantly associated with the distribution of ZHENG (*P* < 0.05). The rs11466285 TT genotype increased the risk of damp heat with toxin (DHT) and deficiency of both *Qi* and *yin* (DQY) compared with obstruction of blood stasis (OBS). The rs884225 AA genotype could increase the risk of DQY and deficiency of both *Qi* and blood (DQB) compared with *yin* deficiency due to stomach heat (YDSH). Parallel comparison among the SNPs and syndrome types revealed that DQB was distinct from YDSH, disharmony between the liver and stomach, stagnation of phlegm muddiness (SPM), OBS, and other syndromes at several SNP loci (*P* < 0.05). The rs11466285 TT and rs884225 AA genotypes exhibit increased risk of DQB compared with OBS and SPM (*P* < 0.05), respectively. In conclusion, the formation of GC ZHENG was related to *EGF*, *TGFA*, and *EGFR* gene polymorphisms.

## 1. Introduction

Gastric cancer (GC) is the second leading cause of cancer-related death worldwide. Most patients present with an advanced stage of the disease, which has a poor outcome. Evidently, there is a need for the development of new tactics for the treatment of this disease [1]. Traditional Chinese medicine (TCM) takes a holistic approach to medicine with emphasis on the integrity of the human body and the relationship between the human and the social and natural environments and provides a theoretical and practical approach to the treatment of GC [2]. TCM therapy, which has been effective in treating GC and improving patient quality of life, is characterized by treatment based on “syndrome differentiation” (also called ZHENG or TCM pattern) [3–5]. Correct TCM syndrome differentiation is the most important principle guiding the prescription of Chinese herbal formulae, and incorrect classification may result in serious consequences [6]. TCM focuses on treating the disease symptoms. Therefore, the diagnostic process mainly includes the gathering of data on the symptoms experienced by the physician. This evidence gathering is done using four manipulations: inspection, auscultation and olfaction, inquiry, and palpation.

The information obtained from syndrome differentiation, including symptoms, pulse feel, and the appearance of the tongue, is often considered to be subjective. Since the tongue is considered in TCM to be an outer manifestation of the spleen and stomach, clinical literature suggests that tongue appearance is valuable for TCM diagnosis of malignant gastrointestinal cancers, such as esophageal cancer, GC, hepatic carcinoma, and colorectal cancer [7, 8]. Aspects of tongue appearance include tongue coating, tongue body, and sublingual veins. TCM medical documents indicate that tongue coating is the most valuable parameter of tongue appearance [9] and plays a role in syndrome differentiation of GC [10]. Our previous results indicated that the expression levels of epidermal growth factor (EGF), transforming growth factor alpha (TGF-*α*), and EGF receptor (EGFR) were closely related to the formation of tongue coating [11, 12]. Therefore, we hypothesized that EGF, TGF-*α*, and EGFR can be correlated with the ZHENG of GC.

EGF and TGF-*α* are members of the EGF super family of cytokines. They function as pleiotropic molecules during many development and pathological processes, such as wound healing and cancer progression. The diverse effects of EGF and TGF-*α* on dermal fibroblasts are initiated by their interaction with EGFR. The EGFR has a high affinity for its ligands, including EGF and TGF-*α*. EGFR is extensively expressed on the basolateral membrane of intestinal epithelial cells and has multiple physiological effects in the response of the gastric mucosa to wounding *in vivo* [13]. Animal experiments show that some compounds in Chinese herbal medicines could improve the quality of experimental gastric ulcer healing by upregulating the expression of EGF and TGF-*α* in the tissue around gastric ulcer [14, 15]. Moreover, *Helicobacter pylori* infection can promote EGFR activation, which has an antiapoptotic effect to protect gastric epithelial cells [16]. These results suggest that moderate activation of the EGFR signal pathway plays an important role in the gastrointestinal mucosal repair processes following injury. However, the overactivation of EGFR participates in several essential tumorigenic mechanisms, such as tumor survival, invasion, angiogenesis, and metastatic spread. Clinical research has observed overexpression of EGFR in numerous human tumors, and several studies have demonstrated that overexpression of EGFR correlates with poor prognosis [17]. In GC, EGFR positivity is considered to be a negative prognostic factor in GC, and biomarker analysis shows that EGFR positivity is associated with poor patient outcomes after curative resection of tumor tissue [18]. EGFR overexpression is correlated with advanced tumor stage and a poor clinical outcome [19]. The EGFR pathway is regulated through the generation of ligands that activate the pathway. Therefore, a considerable amount is known about the mechanisms mediating EGF and TGF-*α* signal transduction [20]. Gene variations (such as single nucleotide polymorphisms (SNPs)) can influence the translation or mRNA degradation [21, 22].

Many studies have suggested that genetic alterations play an important role in the development and progression of GC through gene-environment interactions [23]. In GC, higher levels of EGF, TGF-*α*, and EGFR correlate with advanced tumor stage and a poor clinical outcome [19, 24–26]. Previous studies have reported that the gene variations in *EGF*, *TGFA*, and *EGFR* can lead to deregulation of the EGFR pathway and overexpression of EGF, TGF-*α*, and EGFR proteins [27–30], which are associated with GC and various malignancies [31, 32]. Application of TCM as an adjuvant cancer therapy can enhance quality of life of malignant GC patients and help reduce the adverse effects of chemo- and radiotherapy [33, 34]. ZHENG is an important classification in the subtype for GC TCM therapy. In this study, we hypothesized that genetic variations in *EGF*, *TGFA*, and *EGFR* may affect the formation of ZHENG of GC and form the genetic basis underlying ZHENG. To test this hypothesis, twenty-nine known SNPs in the *EGF*, *TGFA*, and *EGFR* genes were genotyped in a hospital-based population of 387 GC patients broken down into nine types of ZHENG.

## 2. Materials and Methods

### 2.1. Study Subjects

This research protocol was approved by the local ethics committee of Jiangsu Province Hospital of TCM, based on the Declaration of Helsinki. A total of 387 incident GC patients were consecutively recruited from January 2008 to July 2010 in Nanjing, Jiangsu province, eastern China. All subjects were genetically unrelated ethnic Han Chinese. A standard questionnaire was administered by trained interviewers to obtain demographic information and information on related risk factors, including tobacco smoking and alcohol consumption. After signing informed consent documentation, a 3–5 mL venous blood sample was collected from each subject.

### 2.2. Diagnostic Criteria

Diagnoses of all of the patients were confirmed by pathology. Trained interviewers used a uniform questionnaire to collect the TCM diagnostic information from the participants, namely, demographic factors such as age and gender, and known risk factors for GC (such as smoking, drinking, and a family history of digestive tract cancer). The standard criteria used for differentiation of GC ZHENG were as described previously [35]. Nine types of GC ZHENG were used: *Pi Wei Xu Ruo* (spleen and stomach deficiency, SSD), *Wei Re Yin Shang* (*yin* deficiency due to stomach heat, YDSH), *Qi Yin Liang Xu* (deficiency of both *Qi* and *yin*, DQY), *Qi Xue Liang Xu* (deficiency of both *Qi* and blood, DQB), *Gan Wei Bu He* (disharmony between the liver and stomach, DLS), *Shi Re Yun Du* (damp heat with toxin, DHT), *Tan Zhuo Ning Zhi* (stagnation of phlegm muddiness, SPM), *Yu Xue Nei Zu* (obstruction of blood stasis, OBS), and other.

Since many factors may affect the formation TCM syndromes, more than one TCM syndrome was observed in the majority of patients. To ensure a uniform and standard GC ZHENG, the most significant TCM syndromes functioned as units, which were worked out concurrently by two TCM clinical experts. Differentiation criteria for GC are as follows:

SSD: poor appetite, distension of the abdomen, severe distension after eating, epigastric pain with a desire for warmth and pressure, nausea and vomiting, loose stool or defecating with no effort, defecating for a long time, morning diarrhea, lower-extremity edema, listlessness, puffy tongue or teeth imprints on the tongue, whitish tongue coating, thready and weak pulse, or a deep and thready pulse.

YDSH: burning heat, pain after eating, dry mouth, hunger but no desire to eat, dry stool, dysphoria with feverish sensation in the chest, palms, and soles, red or crimson tongue, little or no tongue coating, and a thready and rapid pulse.

DQY: epigastric pain, listlessness, dull complexion, emaciation, shortness of breath after moving, spontaneous perspiration and night sweating, thirst but unwilling to drink, pale tongue with little coating, and a thready and weak pulse.

DQB: emaciation, weakness, low voice, dizziness, pale or yellowish complexion, pale lips and nails, palpitation, shortness of breath, spontaneous perspiration and night sweating, lower-extremity edema, a pale tongue with thin or little coating, and a thready, deep, and weak pulse.

DLS: distended stomach with pain, hypochondriac distention, emotional depression, eructation, acid regurgitation, hiccup, poor appetite, light red or red tongue, thin white or thin yellow tongue coating, and a wiry pulse.

DHT: distended pain and burning heat in the stomach, nausea and vomiting, halitosis, thirst, red tongue with yellowish and greasy coating, and a slippery and rapid pulse.

SPM: distended pain across the abdomen, nausea and vomiting or vomiting of thin and mucous fluid, poor appetite or obstructed sensation after eating, regurgitation of food, tastelessness, no thirst, dizziness, lassitude of the body, loose stool, yellowish complexion and edema, pale tongue with whitish and greasy or slippery coating, and a slippery or moderate to thready pulse.

OBS: stabbing pain or knife-like pain, fixed pain, palpable hard lumps, vomiting with red blood, tarry stool, dark purplish lips and nails, darkish complexion, dark purplish tongue or ecchymosis on the tongue, and an unsmooth pulse.

Other: no obvious syndromes.

### 2.3. Inclusion Criteria

Male and female patients with the following characteristics were included in the study: (a) aged between 20 and 80 years, (b) Han Chinese ethnicity (self-reported), (c) newly histopathologically diagnosed with primary GC, (d) lack of previous malignant tumors in other organs, (e) had not had antitumor therapy before recruitment, including chemotherapy and radiotherapy, and (f) did not have severe heart failure, pulmonary insufficiency, or kidney disease.

### 2.4. Genomic DNA Isolation and Genotyping

After signing informed consent forms, each patient donated 3–5 mL of peripheral blood to be used for genomic DNA extraction. A commercial blood DNA extraction kit (AxyPrep-96 kit, Axygen, CA, USA) was used to extract genomic DNA from the blood samples. Purified DNA samples were stored at −20°C until used for genotyping. Quality of DNA was assessed by agarose gel electrophoresis. The twenty-nine known single nucleotide polymorphisms (SNPs) in the *EGF*, *TGFA*, and *EGFR* genes were searched by the criterion “MAF ≥ 0.05” on the website http://www.ncbi.nlm.nih.gov/snp until July 2010 and are listed as follows: ten SNPs in the *EGF* gene (rs3756261 A/G and rs11568835 A/G in the 5′ near region; rs11568849 A/C, rs11568943 G/A, rs2237051 A/G, and rs11569017 A/T in the nonsynonymous exon region; rs4698803 A/T in the intron region; rs2302135 A/G in the synonymous exon region; rs3733625 A/G in the 3′ untranslated region (UTR); and rs4444903 A/G in the 5′ UTR); eight SNPs in the *TGFA* gene (rs3771527 A/T, rs503314 C/G, rs473698 C/G, rs3732253 C/T, rs538118 A/G, and rs11466285 C/T in the 3′ UTR; rs11466306 A/G in the 3′ near region; and rs2166975 G/A in the synonymous exon region); and eleven SNPs in the *EGFR* gene (rs6965469 C/T and rs884904 A/G in the 5′ near region; rs884225 A/G in the 3′ UTR; rs763317 A/G in the intron region; rs2227983 A/G and rs28384375 T/C in the nonsynonymous exon region; rs17337023 A/T, rs1140475 C/T, rs2293347 G/A, rs2072454 C/T, and rs1050171 G/A in the synonymous exon region).

Polymerase chain reaction-ligation detection reaction (PCR-LDR) was used for genotyping the SNPs, as previously described [29, 36]. In brief, primers were synthesized by Shanghai Sangon Biological Engineering Technology and Services (Shanghai, China). Each set of ligase detection reaction probes comprised one common probe and two discriminating probes for the two types (Table S1 in the Supplementary Matrial available online at http://dx.doi.org/10.1155/2013/731071). After the target DNA sequences were amplified using a multiplex PCR method (Figure S1), the ligation reaction for each subject was carried out in a final volume of 10 *μ*L containing 1 × NEB Taq DNA ligase buffer, 12.5 pmol of each probe mix, 0.05 *μ*L Taq DNA ligase, and 1 *μ*L of multi-PCR product. Probe sequences are shown in Table S2. The fluorescent products of the ligase detection reactions were differentiated by an ABI sequencer 377 (Figure S2). To confirm the accuracy of the PCR-LDR genotyping method, direct DNA sequencing of randomly selected PCR products was performed. The proportion of the sequencing samples was about 5%. The PCR-LDR genotyping results showed complete agreement with the direct DNA sequencing results.

### 2.5. Statistical Analyses

All statistical analyses were conducted using SPSS software, version 16.0 (SPSS Inc., Chicago, IL, USA). The allele and genotype distribution of the SNPs were analyzed by a two-sided *χ* ^2^-test among the nine types of GC ZHENG categories. All *P* values were two-sided, and a *P* value < 0.05 was considered statistically significant.

## 3. Results

### 3.1. Characteristics of the Study Subjects

A total of 387 GC patients were included in the analysis. Gender, age, and ZHENG distribution of subjects are shown in Table 1. The gender proportion and smoking status among the nine syndrome types of GC were significantly different (*χ* ^2^ = 22.342, *P* = 0.004; *χ* ^2^ = 15.844, *P* = 0.045), but no significant differences were observed between age and smoking status (*P* > 0.05).

### 3.2. Genotyping Distribution and Syndrome Types of GC

No statistically significant differences were observed for genotype and allele distributions of the ten SNPs in the *EGF* gene among the nine syndrome types of GC (*P* > 0.05) (Table 2). However, in the *TGFA* and *EGFR* genes, two respective SNPs in the 3′ UTR, rs11466285 and rs884225, were significantly different in genotype distribution among the nine GC syndrome types (*χ* ^2^ = 31.012, *P* = 0.013, and *χ* ^2^ = 29.163, *P* = 0.023) (Tables 3 and 4). The rs11466285 TT genotype distinctly increased the risk of DHT and DQY compared with OBS, and the rs884225 AA genotype could increase the risk of DQY and DQB compared with YDSH. A statistically significant difference was observed in allele distribution, but not the genotype distribution, of the SNP rs884904 A/G in the 5′ near region of the *EGFR* gene among the nine GC syndrome types (*P* > 0.05) (Table 4). Since only the T alleles of SNPs rs28384375 and rs4698803 were detected in this study and the nine types of ZHENG were distinct, a parallel comparison of the genotype and allele distribution of the remaining twenty-seven SNPs between arbitrary pairs of syndrome types linked to polymorphic *EGF*, *TGFA*, and *EGFR* was also conducted. Regarding the relationship between *EGF*, *TGFA*, and *EGFR* gene polymorphisms and GC ZHENG, a parallel comparison of the genotype distribution of the SNPs between either of the two syndrome types linked to polymorphic *TGFA* and *EGFR* was conducted. Fifteen SNPs showed significant differences between random pairs of syndrome types (*P* < 0.05) (Table 5). In these SNPs, four SNPs are in the *EGF* gene, four SNPs are in the *TGFA* gene, and seven SNPs are in the *EGFR* gene. This means that five SNPs (rs3733625, rs3771527, rs3732253, rs11466285, and rs884225) are located in the 3′ UTR, five SNPs (rs11569017, rs2166975, rs17337023, rs1140475, and rs2072454) are located in the exon region, rs763317 is located in the intron region, three SNPs (rs11568835, rs6965469, and rs884904) are located in the 5′ near region, and rs4444903 is located in the 5′ UTR.

More than three SNPs differed among the pairs of syndrome types: DQB versus YDSH, DQB versus DLS, DQB versus SPM, DQB versus OBS, and DQB versus other (*P* < 0.05) (Table 5). First, DQB was significantly different from YDSH in three SNPs (3′ UTR rs884225, 5′ near region rs884904, and exon region rs2072454) (Table 5). Compared with YDSH, the rs884225 AA genotype and A allele and the rs884904 GG genotype and G allele increased the risk of DQB (*P* < 0.05). However, the rs2072454 TT genotype and T allele decreased the risk of DQB (*P* < 0.05) (Table 4). Second, DQB was significantly different from DLS in four SNPs (3′ UTR rs3771527, 5′ near region rs6965469, exon region rs1140475, and intron region rs763317). Compared with DLS, the rs3771527 TT genotype decreased the risk of DQB, the rs6965469 C allele increased the risk of DQB (Table 3), the rs1140475 TC genotype and T allele increased the risk of DQB, and the rs763317 GG genotype and G allele increased the risk of DQB (all *P* < 0.05) (Table 4). Third, DQB was significantly different from SPM in three SNPs (5′ near region rs11568835, exon region rs11569017, and intron region rs763317) (*P* < 0.05). Compared with SPM, the rs11568835 GA genotype increased the risk of DQB though the AA genotype was not detected in DQB. The rs11569017 AA genotype and A allele increased the risk of DQB (Table 2), and the rs763317 A allele decreased the risk of DQB (all *P* < 0.05) (Table 4). Fourth, DQB was significantly different from OBS in three SNPs (5′ UTR rs4444903, 3′ UTR rs884225, and 5′ near region rs884904). Compared with OBS, the rs4444903 A allele increased the risk of DQB (Table 2), the rs884225 AA genotype and A allele increased the risk of DQB, and the rs884904 GG genotype and G allele increased the risk of DQB (all *P* < 0.05) (Table 4). Finally, DQB was significantly different from other syndromes in four SNPs (3′ UTR rs3771527, 3′ UTR rs884225, 5′ near region rs884904, and exon region rs2072454). Compared with other syndromes, the rs3771527 homozygote (TT and AA) decreased the risk of DQB, but the heterozygote (TA) increased the risk of DQB (Table 3). The rs884225 AA genotype and A allele increased the risk of DQB, the rs884904 GG genotype and G allele increased the risk of DQB, and the rs2072454 AA genotype and A allele decreased the risk of DQB (all *P* < 0.05) (Table 4). In addition, the rs1140475 TC genotype significantly increased the risk of DQB compared with SSD, and the rs884225 AA genotype significantly increased the risk of DQB compared with DHT (Table 4). These results demonstrate that DQB was the most different from the other five types of ZHENG (SPM, DLS, YDSH, OBS, and other).

Several SNPs were different among several pairs of syndromes. The SNP rs884225 was different in six pairs of syndrome types (DQB versus YDSH, YDSH versus DQY, DQB versus DHT, DQB versus SPM, DQB versus OBS, and DQB versus other syndromes), rs11466285 was different in five pairs of syndrome types (DQY vs. DLS, DHT vs. DLS, DHT versus SPM, DQY versus OBS, and DHT versus OBS), and rs884904 was different in five pairs of syndrome types (DQB versus YDSH, YDSH versus DQY, DQB versus SPM, DQB vs. OBS, and DQB vs. other syndromes). There were also two pairs of syndrome types in which the SNPs were different for rs11568835, rs3733625, rs11569017, rs3771527, rs2166975, rs1140475, rs763317, and rs2072454. However, there was only one pair of syndrome types in which the SNPs were distinctly different for rs4444903, rs3732253, rs6965469, and rs17337023 (Table 5). These results are consistent with observation that rs884225 and rs11466285 are significantly correlated to the distribution of ZHENG in GC patients (*P* < 0.05).

## 4. Discussion

The gathering of evidence from the four manipulations plays an important role in correct differentiation of ZHENG. However, it is not clear whether congenital defects can influence the formation of ZHENG, which may be essential to classifying the subtypes of GC based on the symptoms. TCM hypothesizes that congenital endowments are determined by genes. However, genes can mutate due to a variety of factors, including environmental variation. Genetic variation in the human genome is an emerging resource for studying cancer, a complex set of diseases characterized by both environmental and genetic contributions. The most common type of sequence variation in the human genome is the SNP [37]. SNPs are the most abundant class of human polymorphisms, which is the main reason medical researchers are so interested in them, despite their simplicity and limited polymorphic content. SNPs can be used as markers to identify genes that underlie complex diseases and to realize the full potential of pharmacogenomics by facilitating analysis of variable responses to drugs. With the improvement of SNP genotyping technologies, a variety of different SNP typing protocols are available for researchers [38]. Several studies have shown that TCM syndromes are associated with gene SNPs, for example the serotonin transporter gene polymorphism and excess of liver *yang* syndrome [39], ABCA1 gene polymorphism and phlegm syndrome and blood stasis syndrome in coronary heart disease [40], kidney-*yang* deficiency syndrome and linkage disequilibrium SNPs [41], liver Qi stagnation syndrome and gene polymorphism of tryptophan hydroxylase and G-protein *β*3 submission in HBC patients [42], AT1R gene polymorphism and ZHENG in essential hypertension [43], and some cytokine gene (*TNFA*, *TGFB1*, and *IL10*) polymorphisms and TCM syndromes in hepatitis B cirrhosis patients [44–46]. However, there are few studies on the relationship between genetic susceptibility and GC ZHENG.

GC is a complex disease with a high mortality rate. The identification of vast numbers of SNPs should enable us to prevent or alleviate the disease by detecting potential disease-susceptibility alleles and diallelic markers [47]. The study of genetic variation could provide future implications for preventive and early intervention strategies. Many studies have suggested that GC development, treatment, and clinical outcome are associated with variations in several genes, including *EGFR* [48, 49], glutathione-S-transferase M1 and T1 [50], p53 [51], E-cadherin(*CDH1*) [52], and cyclooxygenase-2 [53]. We showed that lifestyle (such as meal duration) and clinical examination (such as the status of glutamic pyruvic transaminase) were significantly associated with GC ZHENG, and rs13689 in *CDH1* is correlated with the GC ZHENG type [54]. To probe the genetic traits of the GC ZHENG, we examined the gene polymorphisms in *EGF*, *TGFA*, and *EGFR* in 387 GC patients by ZHENG.

EGF and TGF-*α* induce an equipotent stimulation of proliferation because of their homologous structure and ligation by the same receptor (EGFR). Many reports suggest that EGF and EGFR are critical in cancer progression and their gene polymorphisms are correlated with susceptibility to GC [27, 29, 55]. However, the results are not consistent [31]. Until now, there is no evidence on the relationship between *TGFA* gene polymorphisms and GC risk. In the present study, two polymorphisms, rs11466285 in the *TGFA* gene and rs884225 in the *EGFR* gene, were significantly related to the ZHENG of GC (*χ* ^2^ = 31.012, *P* = 0.013 for rs11466285, and *χ* ^2^ = 29.163, *P* = 0.023 for rs884225). The rs11466285 TT genotype increased the risk of DHT and DQY compared with OBS, and the rs884225 AA genotype increases the risk of DQY and DQB compared with YDSH. In contrast, no direct association was found between ZHENG of GC and *EGF* gene polymorphisms, though several previous studies have reported that the *EGF *+61 (A/G) in the 5′ UTR (SNP rs4444903) is associated with various carcinomas, including GC [56–59]. We found that the rs4444903 A allele increased the risk of DQB compared with OBS (Table 2). The results suggest that DQB is correlated to patients genetically predisposed to GC, while OBS is correlated to patients environmentally predisposed to GC. There may also be a genetic difference between GC risk and GC ZHENG.

Interestingly, the two SNPs correlated with GC (rs11466285, rs884225) are both located in the 3′ UTR (promoter region). Genetic variants in the 3′ UTR may influence the stability of mRNA and, therefore, the function of a gene. Notably, the SNP rs11466285 appeared to confer a substantially greater effect in DQY compared with DLS and OBS (*χ* ^2^ = 9.052, *P* = 0.002 and *χ* ^2^ = 4.241, *P* = 0.039) (Table 5). Moreover, the T allele distinctly increased the probability of DQY compared with DLS and OBS. For the 3′ UTR rs884225 in *EGFR*, the AA genotype significantly increased the risk of DQB and DQY compared with YDSH and decreased the risk of OBS and other compared with DQB (*P* < 0.05). Meanwhile, significant differences in gender proportion and smoking and drinking status were also observed among six GC ZHENG: YDSH, DQY, DLS, DQB, OBS, and other (Table 1). Clinical research shows that TGF-*α* expression in gastric mucosal tissue is significantly positively correlated to ZHENG in patients with chronic gastric disease [60]. Moreover, TCM treatment can decrease the expression of EGFR in gastric mucosal tissue from gastric ulcer patients diagnosed with DLS or syndrome of liver invading the spleen [61]. However, no significant difference was observed between EGF expression and ZHENG in patients with chronic atrophic gastritis (the precursor to GC) [62]. These results suggest that differences in the regulation and gene expression of TGF-*α* and EGFR in the gastric mucosal tissues may underlie molecular markers in the formation of GC ZHENG. TCM theory hypothesizes that DQB and DQY are typical *Xu *ZHENG (deficient syndrome), YDSH and DLS are typical* Shi *ZHENG (excessive syndrome), and OBS is a typical admixture of *Xu *and* Shi *ZHENG. Therefore, SNPs rs11466285 and rs884225 could be genetic markers of *Xu *and* Shi *ZHENG in GC patients, and the formation of TCM ZHENG could be the result of the interaction between genetics and environment.

A clinical investigation with 325 GC patients showed that DLS always occurs in the early stages of GC with a higher proportion in females [63], which is consistent with the present study (Table 1). This result may attributed to the TCM theory that the liver is often constitutional for females, and relieving *Qi* of the liver is an important principle for treating the female patients with GC. Interestingly, distinct differences were observed in several pairs of syndrome types and the results showed that DQB was different from DLS, SPM, OBS, and other (Table 5). This suggests that DQB in GC patients may have a genetic background. The formation of syndrome types in GC, especially DQB, was significantly correlated with polymorphisms in *EGF*, *TGFA*, and *EGFR*.

*Qi-*blood circulation theory is one of the basic theories of TCM. *Qi *is used to describe the refined nutritious substances constituting the human body and maintaining life activities and is also used to describe functions of the *Zang-Fu *organs. TCM theory hypothesizes that the *Zang-Fu* connection is more important in functional entities than anatomical assumptions. The famous therapeutic principle is a classic example. That is, measures must be taken to strengthen the spleen in the treatment of liver disease because liver disease tends to be transmitted to the spleen. The spleen connects with the stomach to form an exterior-interior relationship. Since patients with GC are always feeling emotionally distressed and the liver controls mental and emotional activities, DLS was a common ZHENG in GC TCM differentiation. Relieving liver *Qi* stagnation can be applied to promote the life quality of GC patients [3].

*Qi* deficiency syndrome is one of the main symptoms described by objective physiological phenomena and indexes, including shortness of breath and spontaneous perspiration. Moreover, *Qi* deficiency can lead to decreasing nutrient concentrations in the interstitial fluid which affects the *ex vivo* hematopoietic process and may lead to lower blood volume (blood deficiency) [64]. In the development of GC, the stomach function declines, which affects food digestion, nutrient absorption, and ultimately hematopoietic capacity. Therefore, DQB is often seen in the advanced stages of GC, which results in a lower quality of life, and often has a high rate of relapse and metastasis [63]. Therefore, it may be inferred that the gene polymorphisms of *EGF*, *TGFA*, and *EGFR* correlate to malignant GC. Parallel correlation between the SNPs and ZHENG showed that the SNPs in the functional region (such as the UTR and near region) regulating the gene expression were the most closely related to ZHENG. This is consistent with previous literature [39, 41, 46]. These results suggest that the transcription and regulation of many genes are involved in the formation of ZHENG. TCM, which treats diseases with prescriptions of Chinese herbal formulae guided by differentiation, could be an effective complementary choice for patients with advanced malignant GC. TCM could alleviate the symptoms [65] and promote quality of life [3–5]. In addition, TCM theory on antitumor therapy can enrich the knowledge and understanding of the prophylaxis and treatment of GC and can provide insights into the potential utility of ZHENG [66]. A growing number of technologies are being used to investigate the nature of ZHENG, such as image processing [67] and metabolomics [68], which may contribute to a better understanding of the standardization and material basis of ZHENG.

Several limitations in the present study need to be addressed: (1) the sample size may not have been large enough to detect SNPs with a low variant frequency, such as rs4698803 and rs28384375; (2) the polymorphisms that were investigated were selected based on known SNPs and may not give a comprehensive view of the genetic variability of the *EGF*, *TGFA*, and *EGFR*; and (3) detailed information about the GC cases was not collected, including patient survival, whether the tumors were of the intestinal or diffuse type, whether or not there was metastasis, and the effectiveness of drug therapy.

To summarize, two promoter region SNPs, rs11466285 in *TGFA* and rs884225 in *EGFR*, were significantly associated with GC ZHENG, but no association was found for *EGF*. Parallel comparison among the SNPs and syndrome types revealed that DQB may have a unique genetic basis in the formation of GC ZHENG, and the rs11466285 TT genotype in *TGFA* and rs884225 AA genotype distinctly increased the risk DQB. A larger sample size of each type of GC ZHENG is required to validate these results and to address the underlying mechanisms.

## Supplementary Material

Figure S1. The products of multiplex PCR were detected by agarose gel electrophoresis.
Figure S2. The fluorescent products of LDR were differentiated by ABI sequencer 377 for the twenty-nine SNPs in EGF, TGF-? and EGFR genes.
Table S1. The PCR primer sequences for the twenty-nine loci in the EGF, TGF-? and EGFR gene.
Table S2. Probe sequences of the twenty-nine SNPs in EGF, TGF-? and EGFR gene.

## Figures and Tables

**Table 1:** Common characteristics of patients.

Parameters	Type of ZHENG									*χ* ^2^	*P*
	SSD	YDSH	DQY	DQB	DLS	DHT	SPM	OBS	Other		
Cases											
*n* (%)	61 (15.8)	59 (15.2)	23 (5.9)	49 (12.7)	43 (11.1)	54 (14.0)	41 (10.6)	35 (9.0)	22 (5.7)	—	—
Gender											
Male *n* (%)	41 (67.2)	45 (76.3)	13 (56.5)	31 (63.3)	21 (48.8)	43 (79.6)	31 (75.6)	31 (88.6)	15 (68.2)	22.342	0.004
Female *n* (%)	20 (32.8)	14 (23.7)	10 (43.5)	18 (36.7)	22 (51.2)	11 (20.4)	10 (24.4)	4 (11.4)	7 (31.8)	—	—
Age (mean ± S)											
Total cases	58.3 ± 11.7	61.0 ± 10.6	57.9 ± 13.2	59.8 ± 11.7	59.3 ± 13.2	61.2 ± 12.8	57.3 ± 10.5	60.4 ± 9.3	55.4 ± 13.1	8.472	0.389
Male	61.2 ± 9.3	61.6 ± 9.9	62.1 ± 6.9	60.5 ± 10.9	61.9 ± 12.8	63.0 ± 12.1	59.5 ± 10.2	61.5 ± 9.1	58.3 ± 11.0	4.200	0.839
Female	52.6 ± 14.1	59.0 ± 12.8	52.4 ± 17.4	58.8 ± 13.0	57.0 ± 13.5	57.0 ± 15.0	50.5 ± 8.8	52.5 ± 7.8	49.3 ± 16.1	7.989	0.435
<60	30 (49.2)	29 (49.2)	13 (56.5)	26 (53.1)	23 (53.5)	24 (44.4)	29 (70.7)	17 (48.6)	16 (72.7)	10.802	0.213
>60	31 (50.8)	30 (50.8)	10 (43.5)	23 (46.9)	20 (46.5)	30 (55.6)	12 (29.3)	18 (51.4)	6 (27.3)	—	—
Smoking *n* (%)											
No	33 (54.1)	26 (44.1)	13 (56.5)	28 (57.1)	28 (65.1)	25 (46.3)	23 (56.1)	14 (40.0)	14 (63.6)	9.392	0.310
Yes	28 (45.9)	33 (55.9)	10 (43.5)	21 (42.9)	15 (34.9)	29 (53.7)	18 (43.9)	21 (60.0)	8 (36.4)	—	—
Drinking *n* (%)											
No	29 (47.5)	24 (40.7)	9 (39.1)	34 (69.4)	25 (58.1)	25 (46.3)	16 (39.0)	13 (37.1)	10 (45.5)	15.844	0.045
Yes	32 (52.5)	35 (59.3)	14 (60.9)	15 (30.6)	18 (41.9)	29 (53.7)	25 (61.0)	22 (62.9)	12 (54.5)	—	—

**Table 2:** Relationship between *EGF* gene polymorphisms and the ZHENG of GC.

SNPs	Genotype	Type of ZHENG *n* (%)									*χ* ^2^	*P*
		SSD	YDSH	DQY	DQB	DLS	DHT	SPM	OBS	Other		
rs11568943	GG	37 (60.7)	34 (58.6)	13 (56.5)	31 (64.6)	25 (58.1)	32 (59.3)	17 (42.5)	24 (68.6)	14 (63.6)	15.002	0.524
	GA	21 (34.4)	19 (32.8)	9 (39.1)	14 (29.2)	17 (39.5)	18 (33.3)	23 (57.5)	9 (25.7)	7 (31.8)	—	—
	AA	3 (4.9)	5 (8.6)	1 (4.3)	3 (6.2)	1 (2.3)	4 (7.4)	0 (0.0)	2 (5.7)	1 (4.5)	—	—
	GA/AA	24 (39.3)	24 (41.4)	10 (43.5)	17 (35.4)	18 (41.9)	22 (40.7)	23 (57.5)	11 (31.4)	8 (36.4)	6.791	0.559
	G allele	95 (77.9)	87 (75.0)	35 (76.1)	76 (79.2)	67 (77.9)	82 (75.9)	57 (71.3)	57 (81.4)	35 (79.5)	3.125	0.926
	A allele	27 (22.1)	29 (25.0)	11 (23.9)	20 (20.8)	19 (22.1)	26 (24.1)	23 (28.8)	13 (18.6)	9 (20.5)	—	—

rs3733625	AA	42 (68.9)	36 (62.1)	17 (73.9)	32 (66.7)	34 (79.1)	40 (74.1)	22 (55.0)	26 (74.3)	18 (81.8)	17.171	0.375
	GA	17 (27.9)	21 (36.2)	6 (26.1)	13 (27.1)	8 (18.6)	12 (22.2)	18 (45.0)	8 (22.9)	3 (13.6)	—	—
	GG	2 (3.3)	1 (1.7)	0 (0.0)	3 (6.2)	1 (2.3)	2 (3.7)	0 (0.0)	1 (2.9)	1 (4.5)	—	—
	GA/GG	19 (31.1)	22 (37.9)	6 (26.1)	16 (33.3)	9 (20.9)	14 (25.9)	18 (45.0)	9 (25.7)	4 (18.2)	10.233	0.249
	A allele	101 (82.8)	93 (80.2)	40 (87.0)	77 (80.2)	76 (88.4)	92 (85.2)	62 (77.5)	60 (85.7)	39 (88.6)	6.946	0.542
	G allele	21 (17.2)	23 (19.8)	6 (13.0)	19 (19.8)	10 (11.6)	16 (14.8)	18 (22.5)	10 (14.3)	5 (11.4)	—	—

rs2302135	AA	31 (50.8)	29 (50.9)	9 (39.1)	23 (47.9)	22 (51.2)	27 (50.0)	21 (52.5)	17 (48.6)	14 (63.6)	5.218	0.995
	GA	26 (42.6)	25 (43.9)	12 (52.2)	22 (45.8)	18 (41.9)	24 (44.4)	18 (45.0)	15 (42.9)	6 (27.3)	—	—
	GG	4 (6.6)	3 (5.3)	2 (8.7)	3 (6.2)	3 (7.0)	3 (5.6)	1 (2.5)	3 (8.6)	2 (9.1)	—	—
	GA/GG	30 (49.2)	28 (49.1)	14 (60.9)	25 (52.1)	21 (48.8)	27 (50.0)	19 (47.5)	18 (51.4)	8 (36.4)	2.969	0.936
	A allele	88 (72.1)	83 (72.8)	30 (65.2)	68 (70.8)	62 (72.1)	78 (72.2)	60 (75.0)	49 (70.0)	34 (77.3)	2.26	0.972
	G allele	34 (27.9)	31 (27.2)	16 (34.8)	28 (29.2)	24 (27.9)	30 (27.8)	20 (25.0)	21 (30.0)	10 (22.7)	—	—

rs2237051	AA	24 (39.3)	21 (36.2)	12 (52.2)	19 (39.6)	17 (39.5)	22 (40.7)	19 (47.5)	19 (54.3)	9 (40.9)	15.040	0.522
	GA	29 (47.5)	33 (56.9)	9 (39.1)	26 (54.2)	25 (58.1)	30 (55.6)	18 (45.0)	14 (40.0)	9 (40.9)	—	—
	GG	8 (13.1)	4 (6.9)	2 (8.7)	3 (6.2)	1 (2.3)	2 (3.7)	3 (7.5)	2 (5.7)	4 (18.2)	—	—
	GA/GG	37 (60.7)	37 (63.8)	11 (47.8)	29 (60.4)	26 (60.5)	32 (59.3)	21 (52.5)	16 (45.7)	13 (59.1)	4.875	0.771
	A allele	77 (63.1)	75 (64.7)	33 (71.7)	64 (66.7)	59 (68.6)	74 (68.5)	56 (70.0)	52 (74.3)	27 (61.4)	4.431	0.816
	G allele	45 (36.9)	41 (35.3)	13 (28.3)	32 (33.3)	27 (31.4)	34 (31.5)	24 (30.0)	18 (25.7)	17 (38.6)	—	—

rs11568849	AA	57 (93.4)	57 (98.3)	21 (95.5)	48 (100.0)	42 (100.0)	51 (94.4)	38 (97.4)	35 (100.0)	21 (95.5)	8.744	0.364
	CA	4 (6.6)	1 (1.7)	1 (4.5)	0 (0.0)	0 (0.0)	3 (5.6)	1 (2.6)	0 (0.0)	1 (4.5)	—	—
	A allele	118 (96.7)	115 (99.1)	43 (97.7)	96 (100.0)	84 (100.0)	105 (97.2)	77 (98.7)	70 (100.0)	43 (97.7)	8.616	0.376
	C allele	4 (3.3)	1 (0.9)	1 (2.3)	0 (0.0)	0 (0.0)	3 (2.8)	1 (1.3)	0 (0.0)	1 (2.3)	—	—

rs3756261	AA	40 (65.6)	36 (62.1)	13 (56.5)	32 (66.7)	28 (65.1)	33 (61.1)	18 (45.0)	21 (61.8)	15 (68.2)	13.120	0.664
	GA	17 (27.9)	17 (29.3)	9 (39.1)	13 (27.1)	14 (32.6)	18 (33.3)	21 (52.5)	12 (35.3)	7 (31.8)	—	—
	GG	4 (6.6)	5 (8.6)	1 (4.3)	3 (6.2)	1 (2.3)	3 (5.6)	1 (2.5)	1 (2.9)	0 (0.0)	—	—
	GA/GG	21 (34.4)	22 (37.9)	10 (43.5)	16 (33.3)	15 (34.9)	21 (38.9)	22 (55.0)	13 (38.2)	7 (31.8)	6.479	0.594
	A allele	97 (79.5)	89 (76.7)	35 (76.1)	77 (80.2)	70 (81.4)	84 (77.8)	57 (71.3)	54 (79.4)	37 (84.1)	4.377	0.822
	G allele	25 (20.5)	27 (23.3)	11 (23.9)	19 (19.8)	16 (18.6)	24 (22.2)	23 (28.8)	14 (20.6)	7 (15.9)	—	—

rs4444903	GG	27 (44.3)	22 (38.6)	13 (59.1)	20 (45.5)	21 (50.0)	24 (44.4)	20 (50.0)	21 (61.8)	9 (42.9)	14.518	0.560
	GA	27 (44.3)	31 (54.4)	7 (31.8)	19 (43.2)	19 (45.2)	24 (44.4)	19 (47.5)	12 (35.3)	8 (38.1)	—	—
	AA	7 (11.5)	4 (7.0)	2 (9.1)	5 (11.4)	2 (4.8)	6 (11.1)	1 (2.5)	1 (2.9)	4 (19.0)	—	—
	GA/AA	34 (55.7)	35 (61.4)	9 (40.9)	24 (54.5)	21 (50.0)	30 (55.6)	20 (50.0)	13 (38.2)	12 (57.1)	6.682	0.571
	G allele	81 (66.4)	75 (65.8)	33 (75.0)	59 (67.0)	61 (72.6)	72 (66.7)	59 (73.8)	54 (79.4)	26 (61.9)	7.911	0.442
	A allele	41 (33.6)	39 (34.2)	11 (25.0)	29 (33.0)	23 (27.4)	36 (33.3)	21 (26.3)	14 (20.6)	16 (38.1)	—	—

rs11569017	AA	39 (67.2)	34 (60.7)	13 (61.9)	28 (70.0)	25 (59.5)	34 (64.2)	15 (39.5)	22 (62.9)	15 (68.2)	16.137	0.443
	TA	17 (29.3)	19 (33.9)	8 (38.1)	9 (22.5)	16 (38.1)	16 (30.2)	21 (55.3)	12 (34.3)	5 (22.7)	—	—
	TT	2 (3.4)	3 (5.4)	0 (0.0)	3 (7.5)	1 (2.4)	3 (5.7)	2 (5.3)	1 (2.9)	2 (9.1)	—	—
	TA/TT	19 (32.8)	22 (39.3)	8 (38.1)	12 (30.0)	17 (40.5)	19 (35.8)	23 (60.5)	13 (37.1)	7 (31.8)	10.511	0.231
	A allele	95 (81.9)	87 (77.7)	34 (81.0)	65 (81.3)	66 (78.6)	84 (79.2)	51 (67.1)	56 (80.0)	35 (79.5)	7.348	0.500
	T allele	21 (18.1)	25 (22.3)	8 (19.0)	15 (18.8)	18 (21.4)	22 (20.8)	25 (32.9)	14 (20.0)	9 (20.5)	—	—

rs11568835	GG	43 (72.9)	49 (84.5)	13 (65.0)	30 (71.4)	27 (65.9)	39 (75.0)	31 (77.5)	22 (66.7)	15 (71.4)	15.005	0.524
	GA	15 (25.4)	7 (12.1)	7 (35.0)	12 (28.6)	12 (29.3)	12 (23.1)	7 (17.5)	11 (33.3)	6 (28.6)	—	—
	AA	1 (1.7)	2 (3.4)	0 (0.0)	0 (0.0)	2 (4.9)	1 (1.9)	2 (5.0)	0 (0.0)	0 (0.0)	—	—
	GA/AA	16 (27.1)	9 (15.5)	7 (35.0)	12 (28.6)	14 (34.1)	13 (25.0)	9 (22.5)	11 (33.3)	6 (28.6)	6.894	0.548
	G allele	101 (85.6)	105 (90.5)	33 (82.5)	72 (85.7)	66 (80.5)	90 (86.5)	69 (86.3)	55 (83.3)	36 (85.7)	4.717	0.787
	A allele	17 (14.4)	11 (9.5)	7 (17.5)	12 (14.3)	16 (19.5)	14 (13.5)	11 (13.8)	11 (16.7)	6 (14.3)	—	—

**Table 3:** Relationship between *TGFA* gene polymorphisms and the ZHENG of GC.

SNPs	Genotype	Type of ZHENG *n* (%)									*χ* ^2^	*P*
		SSD	YDSH	DQY	DQB	DLS	DHT	SPM	OBS	Other		
rs3771527	TT	39 (67.2)	37 (68.5)	15 (71.4)	26 (56.5)	32 (80.0)	37 (69.8)	25 (64.1)	22 (64.7)	16 (76.2)	21.067	0.176
	TA	16 (27.6)	17 (31.5)	4 (19.0)	20 (43.5)	8 (20.0)	13 (24.5)	13 (33.3)	11 (32.4)	3 (14.3)	—	—
	AA	3 (5.2)	0 (0.0)	2 (9.5)	0 (0.0)	0 (0.0)	3 (5.7)	1 (2.6)	1 (2.9)	2 (9.5)	—	—
	TA/AA	19 (32.8)	17 (31.5)	6 (28.6)	20 (43.5)	8 (20.0)	16 (30.2)	14 (35.9)	12 (35.3)	5 (23.8)	6.740	0.565
	T allele	94 (81.0)	91 (84.3)	34 (81.0)	72 (78.3)	72 (90.0)	87 (82.1)	63 (80.8)	55 (80.9)	35 (83.3)	5.019	0.756
	A allele	22 (19.0)	17 (15.7)	8 (19.0)	20 (21.7)	8 (10.0)	19 (17.9)	15 (19.2)	13 (19.1)	7 (16.7)	—	—

rs538118	AA	27 (45.8)	24 (41.4)	13 (56.5)	27 (56.2)	24 (58.5)	27 (50.9)	21 (52.5)	17 (48.6)	9 (40.9)	11.900	0.751
	GA	26 (44.1)	28 (48.3)	6 (26.1)	15 (31.2)	15 (36.6)	23 (43.4)	16 (40.0)	15 (42.9)	12 (54.5)	—	—
	GG	6 (10.2)	6 (10.3)	4 (17.4)	6 (12.5)	2 (4.9)	3 (5.7)	3 (7.5)	3 (8.6)	1 (4.5)	—	—
	GA/GG	32 (54.2)	34 (58.6)	10 (43.5)	21 (43.8)	17 (41.5)	26 (49.1)	19 (47.5)	18 (51.4)	13 (59.1)	5.356	0.719
	A allele	80 (67.8)	76 (65.5)	32 (69.6)	69 (71.9)	63 (76.8)	77 (72.6)	58 (72.5)	49 (70.0)	30 (68.2)	3.99	0.858
	G allele	38 (32.2)	40 (34.5)	14 (30.4)	27 (28.1)	19 (23.2)	29 (27.4)	22 (27.5)	21 (30.0)	14 (31.8)	—	—

rs473698	CC	28 (45.9)	25 (43.1)	13 (56.5)	27 (56.2)	24 (55.8)	29 (53.7)	20 (50.0)	18 (52.9)	9 (40.9)	6.733	0.978
	GC	27 (44.3)	28 (48.3)	8 (34.8)	16 (33.3)	16 (37.2)	23 (42.6)	17 (42.5)	14 (41.2)	11 (50.0)	—	—
	GG	6 (9.8)	5 (8.6)	2 (8.7)	5 (10.4)	3 (7.0)	2 (3.7)	3 (7.5)	2 (5.9)	2 (9.1)	—	—
	GC/GG	33 (54.1)	33 (56.9)	10 (43.5)	21 (43.8)	19 (44.2)	25 (46.3)	20 (50.0)	16 (47.1)	13 (59.1)	4.354	0.824
	C allele	83 (68.0)	78 (67.2)	34 (73.9)	70 (72.9)	64 (74.4)	81 (75.0)	57 (71.3)	50 (73.5)	29 (65.9)	3.766	0.878
	G allele	39 (32.0)	38 (32.8)	12 (26.1)	26 (27.1)	22 (25.6)	27 (25.0)	23 (28.8)	18 (26.5)	15 (34.1)	—	—

rs3732253	CC	33 (54.1)	25 (43.1)	10 (43.5)	25 (52.1)	18 (41.9)	19 (35.2)	16 (40.0)	20 (57.1)	11 (50.0)	15.486	0.489
	TC	23 (37.7)	27 (46.6)	8 (34.8)	19 (39.6)	22 (51.2)	31 (57.4)	20 (50.0)	14 (40.0)	8 (36.4)	—	—
	TT	5 (8.2)	6 (10.3)	5 (21.7)	4 (8.3)	3 (7.0)	4 (7.4)	4 (10.0)	1 (2.9)	3 (13.6)	—	—
	TC/TT	28 (45.9)	33 (56.9)	13 (56.5)	23 (47.9)	25 (58.1)	35 (64.8)	24 (60.0)	15 (42.9)	11 (50.0)	7.887	0.445
	C allele	89 (73.0)	77 (66.4)	28 (60.9)	69 (71.9)	58 (67.4)	69 (63.9)	52 (65.0)	54 (77.1)	30 (68.2)	7.097	0.526
	T allele	33 (27.0)	39 (33.6)	18 (39.1)	27 (28.1)	28 (32.6)	39 (36.1)	28 (35.0)	16 (22.9)	14 (31.8)	—	—

rs11466306	GG	33 (54.1)	22 (37.9)	7 (31.8)	25 (52.1)	17 (40.5)	20 (37.0)	18 (45.0)	20 (57.1)	11 (50.0)	15.477	0.490
	GA	23 (37.7)	30 (51.7)	10 (45.5)	18 (37.5)	22 (52.4)	30 (55.6)	18 (45.0)	13 (37.1)	8 (36.4)	—	—
	AA	5 (8.2)	6 (10.3)	5 (22.7)	5 (10.4)	3 (7.1)	4 (7.4)	4 (10.0)	2 (5.7)	3 (13.6)	—	—
	GA/AA	28 (45.9)	36 (62.1)	15 (68.2)	23 (47.9)	25 (59.5)	34 (63.0)	22 (55.0)	15 (42.9)	11 (50.0)	9.743	0.284
	G allele	89 (73.0)	74 (63.8)	24 (54.5)	68 (70.8)	56 (66.7)	70 (64.8)	54 (67.5)	53 (75.7)	30 (68.2)	8.786	0.361
	A allele	33 (27.0)	42 (36.2)	20 (45.5)	28 (29.2)	28 (33.3)	38 (35.2)	26 (32.5)	17 (24.3)	14 (31.8)	—	—

rs11466285	TT	48 (80.0)	48 (84.2)	20 (90.9)	39 (81.2)	27 (67.5)	49 (92.5)	28 (70.0)	22 (62.9)	18 (81.8)	31.012	0.013
	TC	12 (20.0)	9 (15.8)	2 (9.1)	9 (18.8)	13 (32.5)	4 (7.5)	12 (30.0)	12 (34.3)	3 (13.6)	—	—
	CC	0 (0.0)	0 (0.0)	0 (0.0)	0 (0.0)	0 (0.0)	0 (0.0)	0 (0.0)	1 (2.9)	1 (4.5)	—	—
	TC/CC	12 (20.0)	9 (15.8)	2 (9.1)	9 (18.8)	13 (32.5)	4 (7.5)	12 (30.0)	13 (37.1)	4 (18.2)	19.716	0.011
	T allele	108 (90.0)	105 (92.1)	42 (95.5)	87 (90.6)	67 (83.8)	102 (96.2)	68 (85.0)	56 (80.0)	39 (88.6)	18.86	0.016
	C allele	12 (10.0)	9 (7.9)	2 (4.5)	9 (9.4)	13 (16.3)	4 (3.8)	12 (15.0)	14 (20.0)	5 (11.4)	—	—

rs503314	GG	24 (42.9)	20 (36.4)	9 (47.4)	22 (48.9)	21 (53.8)	26 (52.0)	19 (48.7)	16 (47.1)	8 (38.1)	6.720	0.978
	GC	25 (44.6)	28 (50.9)	7 (36.8)	17 (37.8)	15 (38.5)	20 (40.0)	17 (43.6)	15 (44.1)	10 (47.6)	—	—
	CC	7 (12.5)	7 (12.7)	3 (15.8)	6 (13.3)	3 (7.7)	4 (8.0)	3 (7.7)	3 (8.8)	3 (14.3)	—	—
	GC/CC	32 (57.1)	35 (63.6)	10 (52.6)	23 (51.1)	18 (46.2)	24 (48.0)	20 (51.3)	18 (52.9)	13 (61.9)	4.793	0.780
	G allele	73 (65.2)	68 (61.8)	25 (65.8)	61 (67.8)	57 (73.1)	72 (72.0)	55 (70.5)	47 (69.1)	26 (61.9)	4.985	0.759
	C allele	39 (34.8)	42 (38.2)	13 (34.2)	29 (32.2)	21 (26.9)	28 (28.0)	23 (29.5)	21 (30.9)	16 (38.1)	—	—

rs2166975	GG	31 (54.4)	24 (42.1)	9 (42.9)	23 (51.1)	17 (41.5)	20 (38.5)	16 (39.0)	21 (63.6)	10 (45.5)	13.479	0.637
	GA	21 (36.8)	26 (45.6)	7 (33.3)	17 (37.8)	20 (48.8)	26 (50.0)	19 (46.3)	9 (27.3)	8 (36.4)	—	—
	AA	5 (8.8)	7 (12.3)	5 (23.8)	5 (11.1)	4 (9.8)	6 (11.5)	6 (14.6)	3 (9.1)	4 (18.2)	—	—
	GA/AA	26 (45.6)	33 (57.9)	12 (57.1)	22 (48.9)	24 (58.5)	32 (61.5)	25 (61.0)	12 (36.4)	12 (54.5)	8.959	0.346
	G allele	83 (72.8)	74 (64.9)	25 (59.5)	63 (70.0)	54 (65.9)	66 (63.5)	51 (62.2)	51 (77.3)	28 (63.6)	8.268	0.408
	A allele	31 (27.2)	40 (35.1)	17 (40.5)	27 (30.0)	28 (34.1)	38 (36.5)	31 (37.8)	15 (22.7)	16 (36.4)	—	—

**Table 4:** Relationship between *EGFR* gene polymorphisms and the ZHENG of GC.

SNPs	Genotype	Type of ZHENG *n* (%)									*χ* ^2^	*P*
		SSD	YDSH	DQY	DQB	DLS	DHT	SPM	OBS	Other		
rs6965469	CC	40 (65.6)	39 (67.2)	13 (56.5)	37 (77.1)	23 (54.8)	37 (68.5)	25 (62.5)	22 (62.9)	13 (59.1)	11.629	0.769
	TC	19 (31.1)	17 (29.3)	10 (43.5)	10 (20.8)	18 (42.9)	17 (31.5)	14 (35.0)	13 (37.1)	9 (40.9)	—	—
	TT	2 (3.3)	2 (3.4)	0 (0.0)	1 (2.1)	1 (2.4)	0 (0.0)	1 (2.5)	0 (0.0)	0 (0.0)	—	—
	TC/TT	21 (34.4)	19 (32.8)	10 (43.5)	11 (22.9)	19 (45.2)	17 (31.5)	15 (37.5)	13 (37.1)	9 (40.9)	6.692	0.570
	C allele	99 (81.1)	95 (81.9)	36 (78.3)	84 (87.5)	64 (76.2)	91 (84.3)	64 (80.0)	57 (81.4)	35 (79.5)	4.996	0.758
	T allele	23 (18.9)	21 (18.1)	10 (21.7)	12 (12.5)	20 (23.8)	17 (15.7)	16 (20.0)	13 (18.6)	9 (20.5)	—	—

rs17337023	AA	24 (40.7)	12 (20.7)	9 (39.1)	16 (33.3)	14 (32.6)	21 (38.9)	11 (27.5)	10 (28.6)	6 (27.3)	18.596	0.290
	TA	18 (30.5)	35 (60.3)	9 (39.1)	24 (50.0)	19 (44.2)	23 (42.6)	20 (50.0)	16 (45.7)	7 (31.8)	—	—
	TT	17 (28.8)	11 (19.0)	5 (21.7)	8 (16.7)	10 (23.3)	10 (18.5)	9 (22.5)	9 (25.7)	9 (40.9)	—	—
	TA/TT	35 (59.3)	46 (79.3)	14 (60.9)	32 (66.7)	29 (67.4)	33 (61.1)	29 (72.5)	25 (71.4)	16 (72.7)	7.966	0.437
	A allele	66 (55.9)	59 (50.9)	27 (58.7)	56 (58.3)	47 (54.7)	65 (60.2)	42 (52.5)	36 (51.4)	19 (43.2)	5.689	0.682
	T allele	52 (44.1)	57 (49.1)	19 (41.3)	40 (41.7)	39 (45.3)	43 (39.8)	38 (47.5)	34 (48.6)	25 (56.8)	—	—

rs2227983	AA	19 (32.2)	12 (21.1)	8 (34.8)	13 (28.3)	14 (35.0)	19 (36.5)	11 (28.9)	9 (25.7)	4 (19.0)	6.997	0.973
	GA	27 (45.8)	32 (56.1)	11 (47.8)	24 (52.2)	20 (50.0)	24 (46.2)	20 (52.6)	17 (48.6)	12 (57.1)	—	—
	GG	13 (22.0)	13 (22.8)	4 (17.4)	9 (19.6)	6 (15.0)	9 (17.3)	7 (18.4)	9 (25.7)	5 (23.8)	—	—
	GA/GG	40 (67.8)	45 (78.9)	15 (65.2)	33 (71.7)	26 (65.0)	33 (63.5)	27 (71.1)	26 (74.3)	17 (81.0)	5.687	0.682
	A allele	65 (55.1)	56 (49.1)	27 (58.7)	50 (54.3)	48 (60.0)	62 (59.6)	42 (55.3)	35 (50.0)	20 (47.6)	5.137	0.743
	G allele	53 (44.9)	58 (50.9)	19 (41.3)	42 (45.7)	32 (40.0)	42 (40.4)	34 (44.7)	35 (50.0)	22 (52.4)	—	—

rs1140475	CC	60 (98.4)	54 (93.1)	18 (78.3)	41 (85.4)	41 (97.6)	48 (88.9)	36 (90.0)	32 (91.4)	21 (95.5)	14.620	0.067
	TC	1 (1.6)	4 (6.9)	5 (21.7)	7 (14.6)	1 (2.4)	6 (11.1)	4 (10.0)	3 (8.6)	1 (4.5)	—	—
	C allele	121 (99.2)	112 (96.6)	41 (89.1)	89 (92.7)	83 (98.8)	102 (94.4)	76 (95.0)	67 (95.7)	43 (97.7)	13.983	0.082
	T allele	1 (0.8)	4 (3.4)	5 (10.9)	7 (7.3)	1 (1.2)	6 (5.6)	4 (5.0)	3 (4.3)	1 (2.3)	—	—

rs884225	GG	19 (31.1)	23 (39.7)	5 (21.7)	7 (14.6)	14 (32.6)	18 (33.3)	11 (27.5)	13 (37.1)	9 (40.9)	29.163	0.023
	GA	27 (44.3)	29 (50.0)	8 (34.8)	23 (47.9)	18 (41.9)	19 (35.2)	24 (60.0)	17 (48.6)	9 (40.9)	—	—
	AA	15 (24.6)	6 (10.3)	10 (43.5)	18 (37.5)	11 (25.6)	17 (31.5)	5 (12.5)	5 (14.3)	4 (18.2)	—	—
	GA/AA	42 (68.9)	35 (60.3)	18 (78.3)	41 (85.4)	29 (67.4)	36 (66.7)	29 (72.5)	22 (62.9)	13 (59.1)	11.047	0.199
	G allele	65 (53.3)	75 (64.7)	18 (39.1)	37 (38.5)	46 (53.5)	55 (50.9)	46 (57.5)	43 (61.4)	27 (61.4)	21.924	0.005
	A allele	57 (46.7)	41 (35.3)	28 (60.9)	59 (61.5)	40 (46.5)	53 (49.1)	34 (42.5)	27 (38.6)	17 (38.6)	—	—

rs884904	AA	19 (31.1)	23 (39.7)	5 (21.7)	7 (14.6)	14 (32.6)	17 (31.5)	11 (27.5)	13 (37.1)	9 (40.9)	25.536	0.061
	GA	28 (45.9)	27 (46.6)	8 (34.8)	24 (50.0)	18 (41.9)	20 (37.0)	24 (60.0)	17 (48.6)	9 (40.9)	—	—
	GG	14 (23.0)	8 (13.8)	10 (43.5)	17 (35.4)	11 (25.6)	17 (31.5)	5 (12.5)	5 (14.3)	4 (18.2)	—	—
	GA/GG	42 (68.9)	35 (60.3)	18 (78.3)	41 (85.4)	29 (67.4)	37 (68.5)	29 (72.5)	22 (62.9)	13 (59.1)	10.953	0.204
	A allele	66 (54.1)	73 (62.9)	18 (39.1)	38 (39.6)	46 (53.5)	54 (50.0)	46 (57.5)	43 (61.4)	27 (61.4)	19.366	0.013
	G allele	56 (45.9)	43 (37.1)	28 (60.9)	58 (60.4)	40 (46.5)	54 (50.0)	34 (42.5)	27 (38.6)	17 (38.6)	—	—

rs763317	GG	37 (61.7)	36 (62.1)	13 (56.5)	36 (75.0)	20 (46.5)	34 (63.0)	22 (55.0)	22 (62.9)	14 (63.6)	12.275	0.725
	GA	21 (35.0)	20 (34.5)	9 (39.1)	10 (20.8)	21 (48.8)	20 (37.0)	16 (40.0)	12 (34.3)	8 (36.4)	—	—
	AA	2 (3.3)	2 (3.4)	1 (4.3)	2 (4.2)	2 (4.7)	0 (0.0)	2 (5.0)	1 (2.9)	0 (0.0)	—	—
	GA/AA	23 (38.3)	22 (37.9)	10 (43.5)	12 (25.0)	23 (53.5)	20 (37.0)	18 (45.0)	13 (37.1)	8 (36.4)	8.796	0.360
	G allele	95 (79.2)	92 (79.3)	35 (76.1)	82 (85.4)	61 (70.9)	88 (81.5)	60 (75.0)	56 (80.0)	36 (81.8)	7.423	0.492
	A allele	25 (20.8)	24 (20.7)	11 (23.9)	14 (14.6)	25 (29.1)	20 (18.5)	20 (25.0)	14 (20.0)	8 (18.2)	—	—

rs2293347	GG	28 (47.5)	37 (64.9)	10 (43.5)	21 (45.7)	23 (54.8)	28 (52.8)	22 (56.4)	21 (61.8)	13 (61.9)	17.979	0.325
	GA	26 (44.1)	19 (33.3)	10 (43.5)	22 (47.8)	13 (31.0)	17 (32.1)	16 (41.0)	11 (32.4)	6 (28.6)	—	—
	AA	5 (8.5)	1 (1.8)	3 (13.0)	3 (6.5)	6 (14.3)	8 (15.1)	1 (2.6)	2 (5.9)	2 (9.5)	—	—
	GA/AA	31 (52.5)	20 (35.1)	13 (56.5)	25 (54.3)	19 (45.2)	25 (47.2)	17 (43.6)	13 (38.2)	8 (38.1)	7.544	0.479
	G allele	82 (69.5)	93 (81.6)	30 (65.2)	64 (69.6)	59 (70.2)	73 (68.9)	60 (76.9)	53 (77.9)	32 (76.2)	9.868	0.274
	A allele	36 (30.5)	21 (18.4)	16 (34.8)	28 (30.4)	25 (29.8)	33 (31.1)	18 (23.1)	15 (22.1)	10 (23.8)	—	—

rs2072454	CC	20 (32.8)	14 (24.1)	10 (43.5)	24 (50.0)	13 (30.2)	20 (37.0)	12 (30.0)	12 (34.3)	5 (22.7)	15.446	0.492
	TC	27 (44.3)	31 (53.4)	8 (34.8)	19 (39.6)	21 (48.8)	24 (44.4)	22 (55.0)	18 (51.4)	10 (45.5)	—	—
	TT	14 (23.0)	13 (22.4)	5 (21.7)	5 (10.4)	9 (20.9)	10 (18.5)	6 (15.0)	5 (14.3)	7 (31.8)	—	—
	TC/TT	41 (67.2)	44 (75.9)	13 (56.5)	24 (50.0)	30 (69.8)	34 (63.0)	28 (70.0)	23 (65.7)	17 (77.3)	10.996	0.202
	C allele	67 (54.9)	59 (50.9)	28 (60.9)	67 (69.8)	47 (54.7)	64 (59.3)	46 (57.5)	42 (60.0)	20 (45.5)	11.759	0.162
	T allele	55 (45.1)	57 (49.1)	18 (39.1)	29 (30.2)	39 (45.3)	44 (40.7)	34 (42.5)	28 (40.0)	24 (54.5)	—	—

rs1050171	GG	46 (79.3)	42 (76.4)	16 (76.2)	27 (65.9)	31 (73.8)	37 (69.8)	27 (71.1)	27 (77.1)	16 (72.7)	6.461	0.982
	GA	12 (20.7)	10 (18.2)	4 (19.0)	12 (29.3)	10 (23.8)	14 (26.4)	10 (26.3)	7 (20.0)	5 (22.7)	—	—
	AA	0 (0.0)	3 (5.5)	1 (4.8)	2 (4.9)	1 (2.4)	2 (3.8)	1 (2.6)	1 (2.9)	1 (4.5)	—	—
	GA/AA	12 (20.7)	13 (23.6)	5 (23.8)	14 (34.1)	11 (26.2)	16 (30.2)	11 (28.9)	8 (22.9)	6 (27.3)	3.288	0.915
	G allele	104 (89.7)	94 (85.5)	36 (85.7)	66 (80.5)	72 (85.7)	88 (83.0)	64 (84.2)	61 (87.1)	37 (84.1)	4.015	0.856
	A allele	12 (10.3)	16 (14.5)	6 (14.3)	16 (19.5)	12 (14.3)	18 (17.0)	12 (15.8)	9 (12.9)	7 (15.9)	—	—

**Table 5:** Parallel comparison of genotype distribution of *EGF*, *TGFA*, and *EGFR* gene SNPs between the arbitrary two types of ZHENG.

Type of ZHENG	*χ* ^2^ (*P*)							
	YDSH	DQY	DQB	DLS	DHT	SPM	OBS	Other
SSD	10.731 (0.005)^10a^ 5.486 (0.019)^10b^	10.174 (0.001)^11a^	6.618 (0.010)^11a^	—	4.495 (0.034)^11a^	—	—	—

YDSH	—	11.517 (0.003)^12a^ 8.588 (0.014)^13a^	—	—	—	—	—	—

DQY	—	—	—	9.502 (0.002)^7a^ 6.647 (0.010)^11a^	—	—	4.241 (0.039)^7a^ 6.247 (0.044)^12a^ 6.247 (0.044)^13a^	—

DQB	14.410 (0.001)^12a^ 8.136 (0.004)^12b^ 11.105 (0.004)^13a^ 8.136 (0.004)^13b^ 8.197 (0.017)^15a^ 7.639 (0.006)^15b^	—	—	5.371 (0.020)^5a^ 5.022 (0.025)^9b^ 4.118 (0.042)^11a^ 4.128 (0.042)^11b^ 8.225 (0.016)^14a^ 7.777 (0.005)^14b^	4.828 (0.028)^12a^	8.616 (0.013)^1a^ 4.292 (0.038)^1b^ 8.885 (0.012)^4a^ 7.341 (0.007)^4b^ 3.884 (0.049)^14b^	4.586 (0.032)^3b^ 8.213 (0.016)^12a^ 5.632 (0.018)^12b^ 7.693 (0.021)^13a^ 5.632 (0.018)^13b^	8.850 (0.012)^5a^ 6.527 (0.038)^12a^ 5.929 (0.015)^12b^ 6.332 (0.042)^13a^ 5.929 (0.015)^13b^ 6.865 (0.032)^15a^ 4.624 (0.032)^15b^

DLS	—	—	—	—	—	7.319 (0.026)^2a^ 5.470 (0.019)^2b^	—	—

DHT	—	—	—	9.502 (0.002)^7a^	—	8.068 (0.005)^7a^	4.159 (0.041)^6b^ 12.092 (0.002)^7a^ 11.846 (0.001)^7b^ 5.124 (0.024)^8b^	—

SPM	—	—	—	—	—	—	9.027 (0.011)^1a^ 5.120 (0.024)^1b^ 4.430 (0.035)^8b^	7.523 (0.023)^2a^ 4.459 (0.035)^2b^ 6.007 (0.050)^4a^ 4.593 (0.032)^4b^

^1^rs11568835, ^2^rs3733625, ^3^rs4444903, and ^4^rs11569017 are located in the *EGF* gene; ^5^rs3771527, ^6^rs3732253, ^7^rs11466285, and ^8^rs2166975 are located in the *TGFA* gene; ^9^rs6965469, ^10^rs17337023,^ 11^rs1140475, ^12^rs884225, ^13^rs884904, ^14^rs763317, and ^15^rs2072454 are located in the *EGFR* gene. Namely, ^2^rs3733625, ^5^rs3771527, ^6^rs3732253, ^7^rs11466285, and ^12^rs884225 are located in the 3′ UTR; ^4^rs11569017, ^8^rs2166975, ^10^rs17337023, ^11^rs1140475, and ^15^rs2072454 are located in the exon region; ^14^rs763317 is located in intron region; ^1^rs11568835, ^9^rs6965469, and ^13^rs884904 are located in the 5′ near region; ^3^rs4444903 is located in the 5′ UTR.

^a^Comparison of the three genotypes; ^b^comparison of wild genotype and mutant genotype.
