# Supplementary material for: Relationship between EGF, TGFA, and EGFR Gene Polymorphisms and Traditional Chinese Medicine ZHENG in Gastric Cancer
Source: Evid Based Complement Alternat Med. 2013 Dec 16;2013:731071. doi: 10.1155/2013/731071 (PMC3876898; doi:10.1155/2013/731071)
Supplement: Supplementary file 1 — Figure S1. The products of multiplex PCR were detected by agarose gel electrophoresis. Figure S2. The fluorescent products of LDR were differentiated by ABI sequencer 377 for the twenty-nine SNPs in EGF, TGF-? and EGFR genes. Table S1. The PCR primer sequences for the twenty-nine loci in the EGF, TGF-? and EGFR gene. Table S2. Probe sequences of the twenty-nine SNPs in EGF, TGF-? and EGFR gene. [file 731071.f1.pdf]

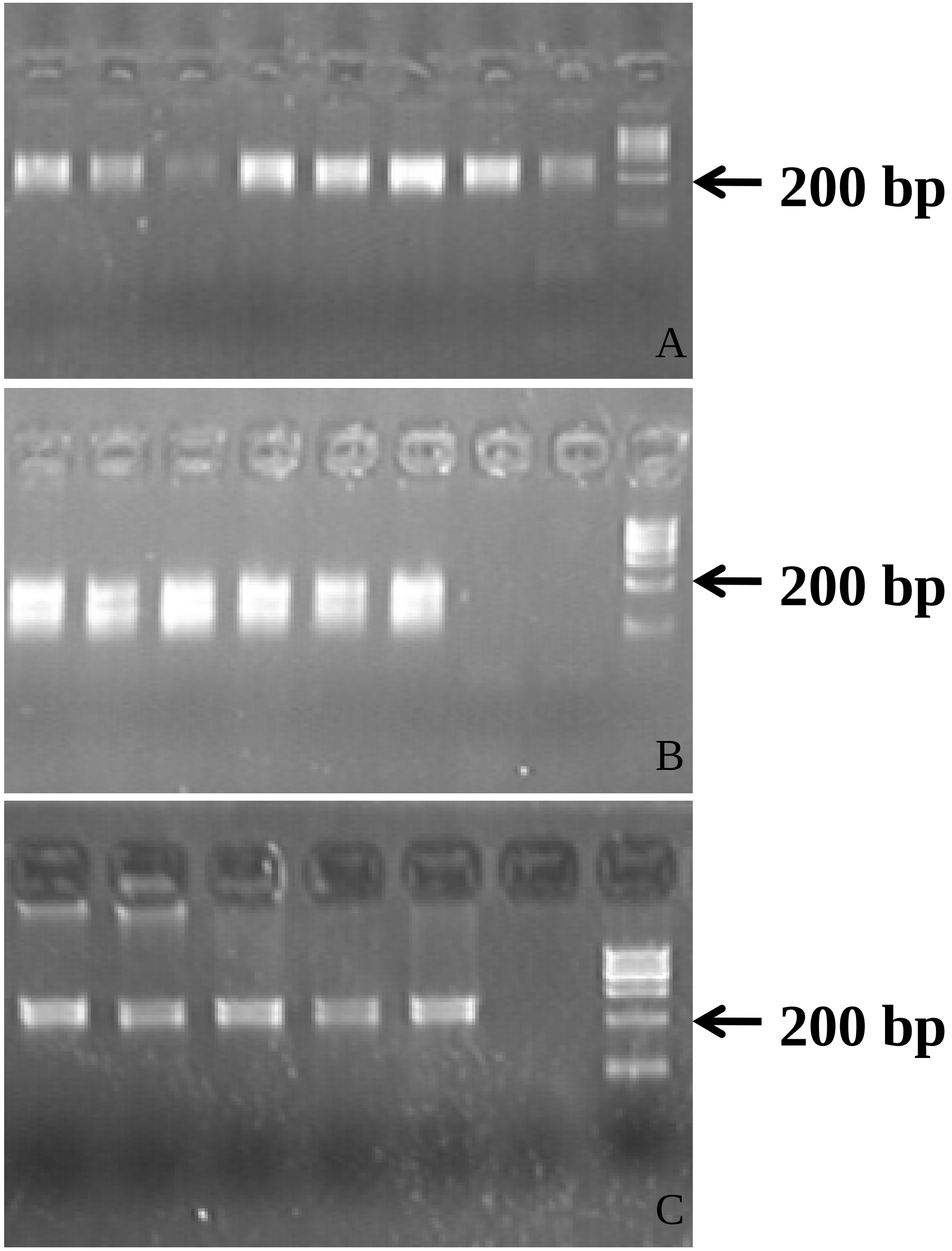

Figure S1 Products of multiplex PCR detected by agarose gel electrophoresis. (A) Products include the SNPs in the *EGF* gene. (B) The products include the SNPs in the *TGFA* gene. (C) The products include the SNPs in the *EGFR* gene.

Gene	SNPs	Typical differential peaks		
		Heterozygous status	Homozygous status	
EGF	rs3756261<br>A/G<br>118/120	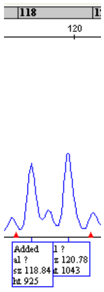	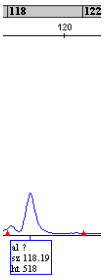	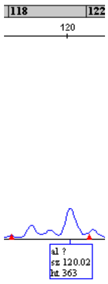
	rs11568835<br>A/G<br>78/80	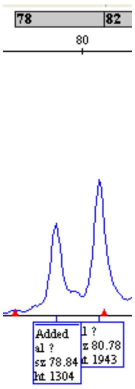	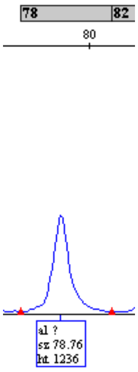	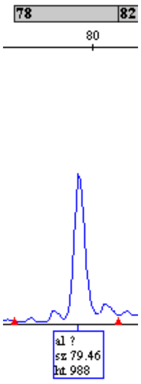
	rs11568849<br>A/C<br>114/116	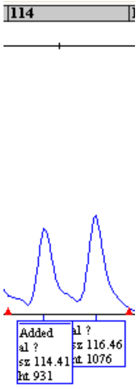	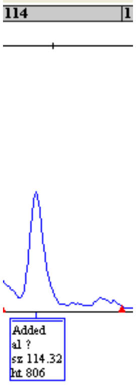	N
	rs11568943<br>A/G<br>86/88	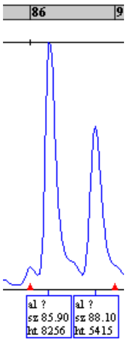	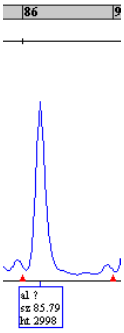	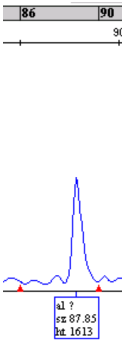

rs2237051
A/G
114/116

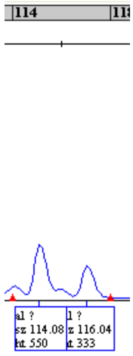

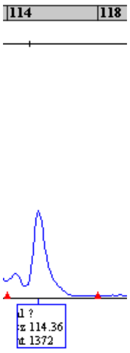

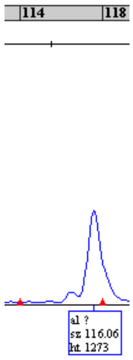

---

rs11569017
A/T
86/88

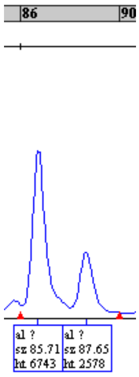

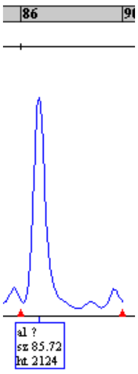

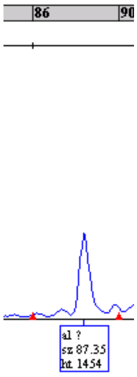

---

rs4698803
A/T
118/120

N

N

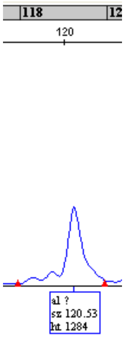

---

rs2302135
A/G
110/112

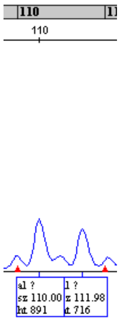

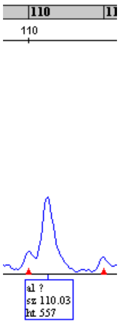

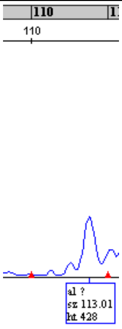

rs3733625
A/G
102/104

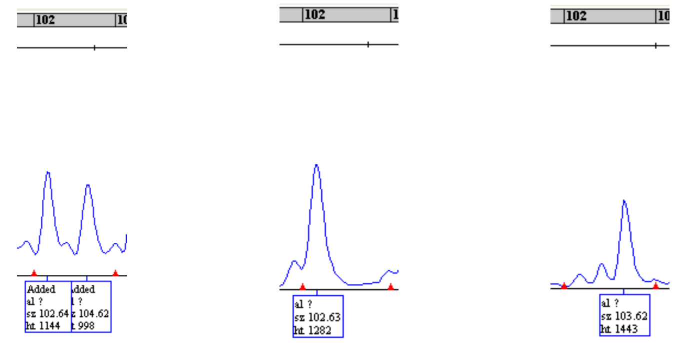

rs4444903
A/G
82/84

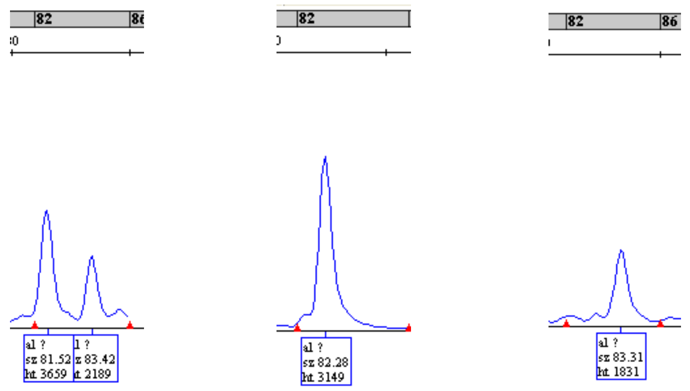

TGFA

rs3771527
A/T
78/80

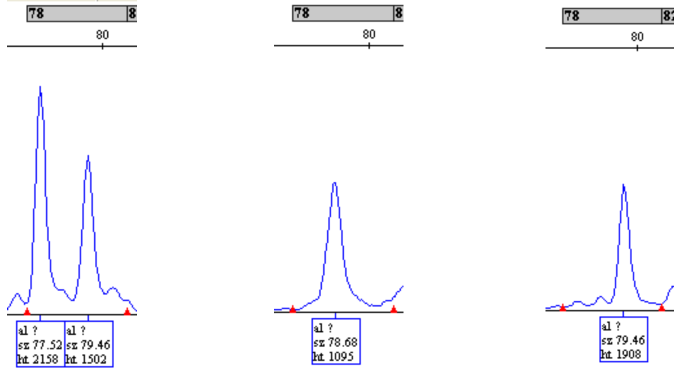

rs473698
C/G
78/80

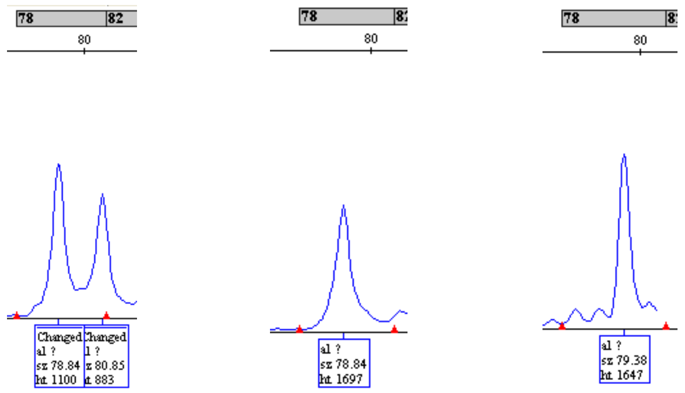

rs3732253
C/T
94/96

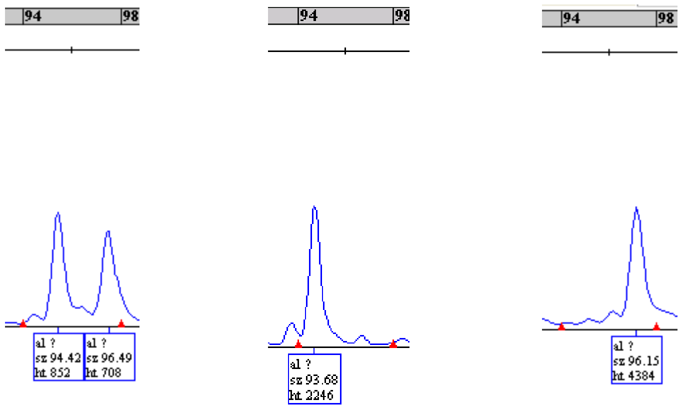

---

rs538118
A/G
90/92

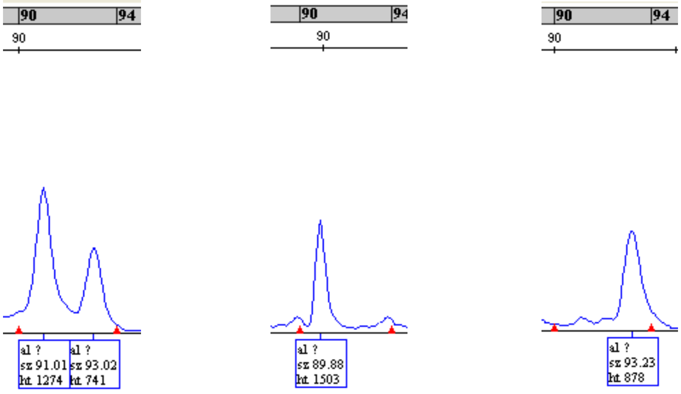

---

rs11466285
102/104
C/T

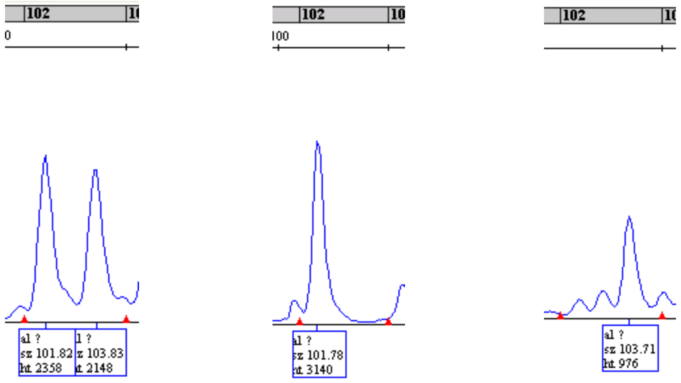

---

rs11466306
98/100
A/G

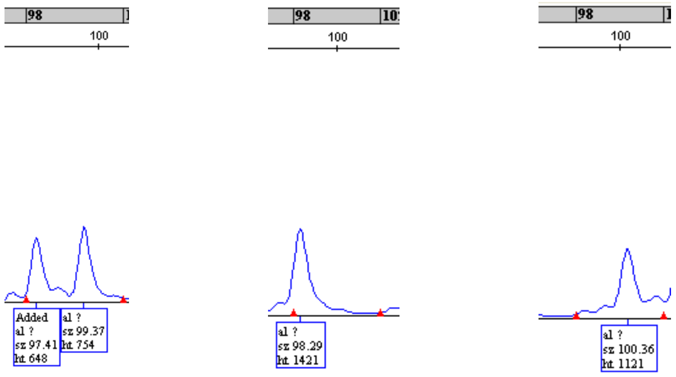

rs2166975
A/G
110/112

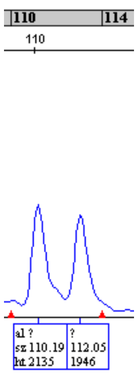

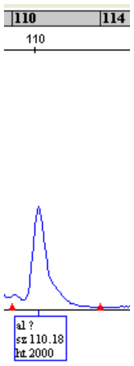

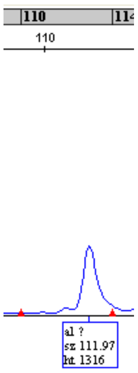

EGFR

rs2227983
A/G
98/100

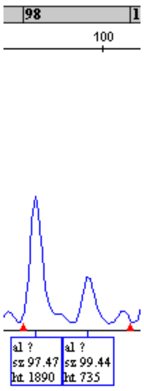

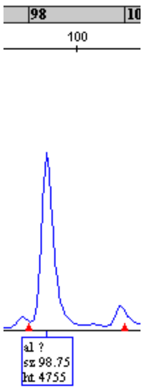

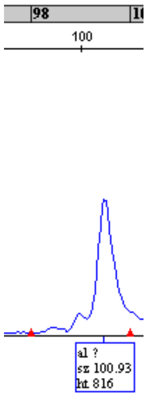

rs17337023
A/T
86/88

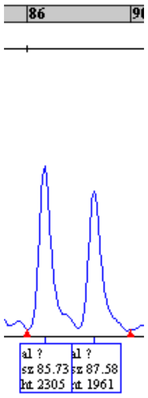

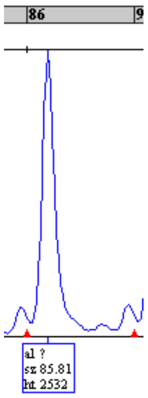

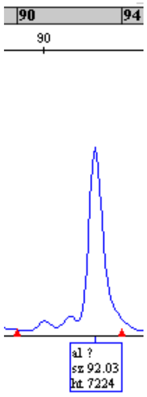

rs1140475
C/T
102/104

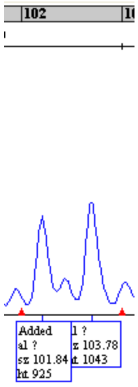

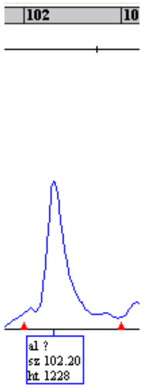

N

rs2293347<br>A/G<br>118/120	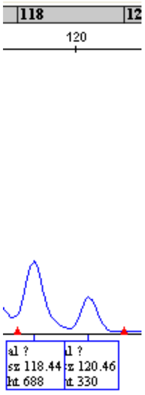	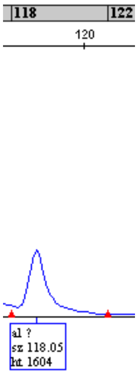	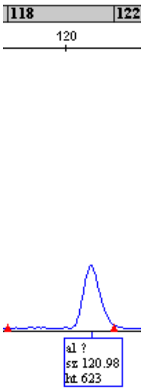
rs2072454<br>C/T<br>106/108	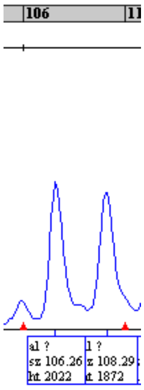	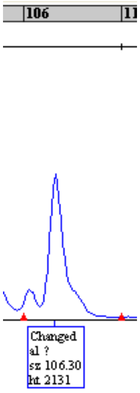	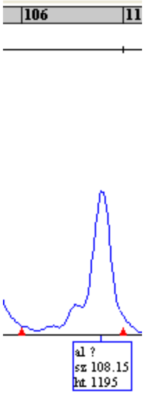
rs28384375<br>C/T<br>90/92	N	N	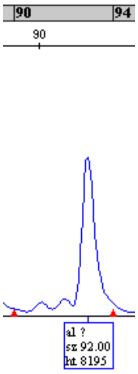
rs1050171<br>A/G<br>94/96	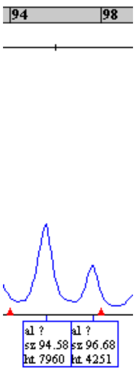	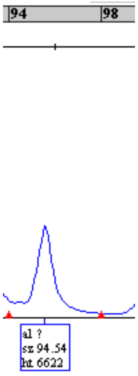	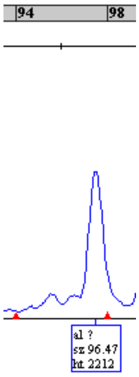

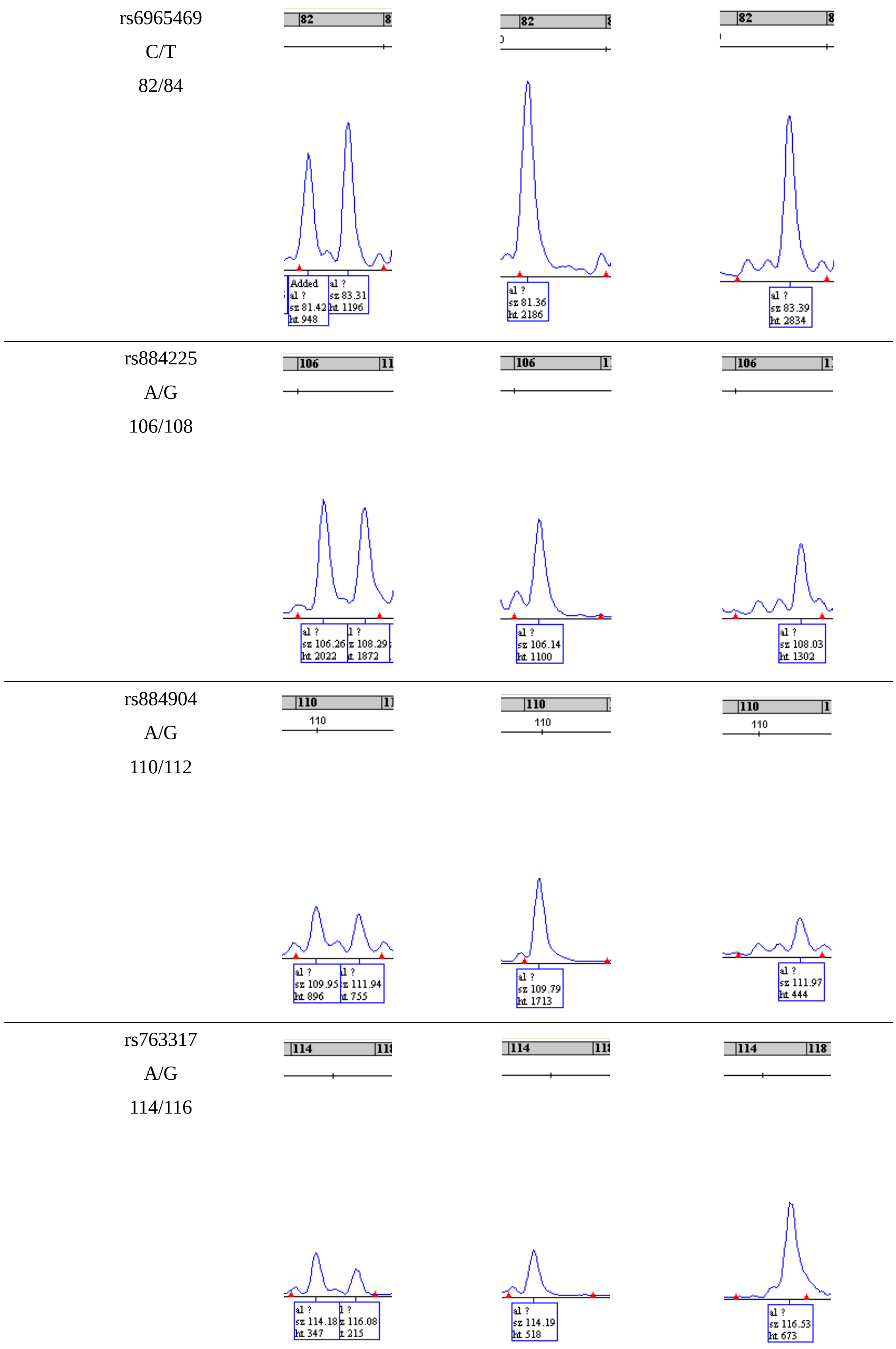

Figure S2 Differentiated results of fluorescent products of LDR by ABI sequencer 377 for the twenty-nine SNPs in *EGF*, *TGFA* and *EGFR*

Table S1 PCR primer sequences for the twenty-nine SNPs in *EGF*, *TGFA*, and *EGFR*

Gene	SNPs	Forward	Reverse	Length(bp)
EGF	rs3756261	GCAGATGCTATGGCTGATGA	GAAGTGTGATCTGCCCACCT	211
	rs11568835	GCAAACCTTTTTCCAACCAA	AACCTGCACTGACTCTTCGAG	240
	rs11568849	ATGCTCCAGCAAAATCAAGC	AGACCTGGAGCACCTTTTCA	216
	rs11568943	TCCACGCAATGTGTCTGAAT	TCAATTTTTAGATGTCACTGAG CAA	185
	rs2237051	TTCAAGCCTTGTCCTTTCGT	GTTTTGCCAATGGATGAACC	243
	rs11569017	TGCAAAAAGAGGCTTGGAAC	TTCAAAATCAGCAAAAGCATA AA	196
	rs4698803	TCTTCCAAAGGCTGTCCAAC	TCACTGTAGACCACCCACCA	231
	rs2302135	TTCAAGCCTTGTCCTTTCGT	GTTTTGCCAATGGATGAACC	243
	rs3733625	CACGCCAATGAGGAGTTAAA	CAAATTGGGACAACAGTGCTT	249
	rs4444903	CATTTGCAAACAGAGGCTCA	TGCTCTGGCTGACTTCACTG	250
TGFA	rs3771527	CCTTACTCTGATGTCTGTATAT GTTGC	TCATTCCTTCATCCTTCCAAA	241
	rs473698	TCTCCTGAGCCATTGGAAAC	TGGCAATGGTAGTCTTTGGTT	212
	rs3732253	TGACCCCCTAGCAACGTAGT	CTCAAGTCTTCGCAGGGAAC	249
	rs538118	GCATTTCCTCACATAAGGAGT TT	TCACCTCCACTATGTGGGTCT	215
	rs11466285	TGGGGAAGAAAGTGAAGGAG	TGTAGAAGTCTGAAGGGGTTT T	250
	rs11466306	TCTCTGAAGCTCTTCCCAGTG	AGGTCTGGAACCATGCTTTG	243
	rs2166975	ATCTCTGGCAGTGCTGTCCT	AATTTGGACTCGGGCCTAAC	231
EGFR	rs2227983	TGCTGTGACCCACTCTGTCT	CAACGCAAGGGGATTAAAG	194
	rs17337023	ACCACCAATCCAACATCCAG	CCACAGCAGTGTGGTCATTC	250
	rs1140475	GAAGCAAATTGCCCAAGACT	AGGCGTTCTCCTTTCTCCAG	237

rs2293347	AGAGAGCTCAGGAGGGGAGT	TAGCATCTCTACGGGCCATT	216
rs2072454	AAAGAGTGCTCACCGCAGTT	CATAGGAGCTGGAGGCAGAG	213
rs28384375	GCATGAACATTTTTCTCCACCT	CTCACCCGTAGGTGCAGTTT	195
rs1050171	CTCCAGGAAGCCTACGTGAT	TTATCTCCCCTCCCCGTATC	230
rs6965469	TCTGTTCCCTGGAATCCATC	TTTGAGTGCCCAAAAAGACA	196
rs884225	TTGGTCCAAATGCTGATGAA	GATGGACCAGTGGTTTCCAG	180
rs884904	GGCTGAGGTCAGTCACCCTA	TTTCTGCATGTCCTGAGGTG	222
rs763317	TCCTTCAGCAAAACCCTCAG	GGACTCCAGTCCAATTTTTCA	218

Table S2 Probe sequences of the twenty-nine SNPs in *EGF*, *TGFA*, and *EGFR*

Gene	SNPs	Allele	Probe sequence (5' - 3')	LDR length
EGF	rs3756261	For LDR	P-AAATCCTTCCCAGTTGGGTAGCTCTTTTTTTTTTTTTTTTTTTTTTTTTTTTTTTTTTT-FAM	
		A specific	TTTTTTTTTTTTTTTTTTTTTTTTTTTTTTTTTTATATAAACTTATTAAATATCTCTGT	118 bp
		G specific	TTTTTTTTTTTTTTTTTTTTTTTTTTTTTTTTTTTTATATAAACTTATTAAATATCTCTGC	120 bp
	rs11568835	For LDR	P-CAGATGACAGCAATGGAAGGGGCTTTTTTTTTTTTTTTT-FAM	
		A specific	TTTTTTTTTTTTTTTCAGCACAATACATTGCCAGCATCT	78 bp
		G specific	TTTTTTTTTTTTTTTTTCAGCACAATACATTGCCAGCATCC	80 bp
	rs11568849	For LDR	P-AAATTTTGAAACTACTGGCAACAGATTTTTTTTTTTTTTTTTTTTTTTTTTTTTTTT-FAM	
		A specific	TTTTTTTTTTTTTTTTTTTTTTTTTTTTTTTTGCGGTGCTGAGAGACTAACAAAACT	114 bp
		C specific	TTTTTTTTTTTTTTTTTTTTTTTTTTTTTTTTTTGCGGTGCTGAGAGACTAACAAAACG	116 bp
	rs11568943	For LDR	P-TCTCAAGCACTGAGCCTTCAGGACATTTTTTTTTTTTTTTTTT-FAM	
		A specific	TTTTTTTTTTTTTTTTTTTCACCGCTACATGTTTTCCCATCTT	86 bp
		G specific	TTTTTTTTTTTTTTTTTTTTTCACCGCTACATGTTTTCCCATCTC	88 bp
	rs2237051	For LDR	P-ATTACTGATGGCATAGCCCAATCTGTTTTTTTTTTTTTTTTTTTTTTTTTTTTTTTT-FAM	
		A specific	TTTTTTTTTTTTTTTTTTTTTTTTTTTTTTTTTTTGCCAGTCCTCTTGTTTACTCTT	114 bp
		G specific	TTTTTTTTTTTTTTTTTTTTTTTTTTTTTTTTTTTTTGCCAGTCCTCTTGTTTACTCTC	116 bp
	rs11569017	For LDR	P-CCAGAGCCAGACACGTTTTCCCATCTTTTTTTTTTTTTTTTTT-FAM	
		A specific	TTTTTTTTTTTTTTTTTTTTACCTGCCAACAGCTGATGACCAT	86 bp
		T specific	TTTTTTTTTTTTTTTTTTTTTTACCTGCCAACAGCTGATGACCAA	88 bp
	rs4698803	For LDR	P-CCCCCAGTTGGCACTCATCAATATCTTTTTTTTTTTTTTTTTTTTTTTTTTTTTTTTTT-FAM	
		A specific	TTTTTTTTTTTTTTTTTTTTTTTTTTTTTTTTTTCTGGCATTCTCTCCACAGCTGTGCT	118 bp
		T specific	TTTTTTTTTTTTTTTTTTTTTTTTTTTTTTTTTTTTCTGGCATTCTCTCCACAGCTGTGCA	120 bp
	rs2302135	For LDR	P-GCTACAGCAAATGGGTGACCTGGAATTTTTTTTTTTTTTTTTTTTTTTTTTTTTT-FAM	
		A specific	TTTTTTTTTTTTTTTTTTTTTTTTTTTTTTGAACCACACATAATCCTCAAACACT	110 bp
		G specific	TTTTTTTTTTTTTTTTTTTTTTTTTTTTTTTTGAACCACACATAATCCTCAAACACC	112 bp
	rs3733625	For LDR	P-CTGGACAGAACATTTATATCAGTTTTTTTTTTTTTTTTTTTTTTTTTTTTT-FAM	

		A specific	TTTTTTTTTTTTTTTTTTTTTTTTTTTCAGTTACAAAGTAATTTCTTTGAT	102 bp
		G specific	TTTTTTTTTTTTTTTTTTTTTTTTTTTTTCAGTTACAAAGTAATTTCTTTGAC	104 bp
	rs4444903	For LDR	P-ACAACCCTTGGATTGGGGCTGAAAGTTTTTTTTTTTTTTTT-FAM	
		A specific	TTTTTTTTTTTTTTTTGAAGAACTGATGGAAAGTTCCAGCT	82 bp
		G specific	TTTTTTTTTTTTTTTTTTGAAGAACTGATGGAAAGTTCCAGCC	84 bp
TGFA	rs3771527	For LDR	P-GAAATCAAAATTAACTTACCACACATTTTTTTTTTTTTT-FAM	
		A specific	TTTTTTTTTTTTTTCTGCTAATCACATTAACACATTACT	78 bp
		T specific	TTTTTTTTTTTTTTTTCTGCTAATCACATTAACACATTACA	80 bp
	rs473698	For LDR	P-AATTGTTCACTACAATTAGAGAATCTTTTTTTTTTTTTT-FAM	
		C specific	TTTTTTTTTTTTTTGTGTTTCCTTGCCCTTTAGAAAATG	78 bp
		G specific	TTTTTTTTTTTTTTTTGTGTTTCCTTGCCCTTTAGAAAATC	80 bp
	rs3732253	For LDR	P-TTGATTGGTCTCTAAGCAGGATGCTTTTTTTTTTTTTTTTTTTTTT-FAM	
		C specific	TTTTTTTTTTTTTTTTTTTTTTATAGAGGTCTAAAAACCAGTGTCCG	94 bp
		T specific	TTTTTTTTTTTTTTTTTTTTTTTTATAGAGGTCTAAAAACCAGTGTCCA	96 bp
	rs538118	For LDR	P-GGGGCCTGCCTGTGAGAAACAGTGGTTTTTTTTTTTTTTTTTTTT-FAM	
		A specific	TTTTTTTTTTTTTTTTTTTTGAGCATCTGGGTCCATCTGACCCAT	90 bp
		G specific	TTTTTTTTTTTTTTTTTTTTTTGAGCATCTGGGTCCATCTGACCCAC	92 bp
	rs11466285	For LDR	P-TGGAAGATGTTGGGCTGGTTGAGGGTTTTTTTTTTTTTTTTTTTTTTTTTT-FAM	
		C specific	TTTTTTTTTTTTTTTTTTTTTTTTTTGATGGTCTTCAATGTCATGTGTCCG	102 bp
		T specific	TTTTTTTTTTTTTTTTTTTTTTTTTTTTGATGGTCTTCAATGTCATGTGTCCA	104 bp
	rs11466306	For LDR	P-CTGCAGTGGCAGAGGTTGTATATATTTTTTTTTTTTTTTTTTTTTTTTT-FAM	
		A specific	TTTTTTTTTTTTTTTTTTTTTTTTTGTCTTCTCGACCAGATTTCAATTT	98 bp
		G specific	TTTTTTTTTTTTTTTTTTTTTTTTTTTGTCTTCTCGACCAGATTTCAATTC	100 bp
	rs2166975	For LDR	P-ACCACTGGCAGGAAGGAAAATTTTTTTTTTTTTTTTTTTTTTTTTTTTTTTTTTTT-FAM	
		C specific	TTTTTTTTTTTTTTTTTTTTTTTTTTTTTTCAAACTCCTCCTCTGGGCTCTTCAG	110 bp
		T specific	TTTTTTTTTTTTTTTTTTTTTTTTTTTTTTTTCAAACTCCTCCTCTGGGCTCTTCAA	112 bp
EGFR	rs2227983	For LDR	P-TGGGCTCCGGGCCCCAGCAGCCCTCTTTTTTTTTTTTTTTTTTTTTTTT-FAM	
		A specific	TTTTTTTTTTTTTTTTTTTTTTTTACATTCCGGCAAGAGACGCAGTCCT	98 bp
		G specific	TTTTTTTTTTTTTTTTTTTTTTTTTTACATTCCGGCAAGAGACGCAGTCCC	100 bp

rs17337023	For LDR	P-GTGCATCTGTAGGAAGTGAAAGAGATTTTTTTTTTTTTTTT-FAM	
	A specific	TTTTTTTTTTTTTTTTTTTGGACAGCCTTCAAGACCTGGCCCT	86 bp
	T specific	TTTTTTTTTTTTTTTTTTTTTGGACAGCCTTCAAGACCTGGCCCA	88 bp
rs1140475	For LDR	P-GTCACCCCTGAGAGGATGAAGCAAGTTTTTTTTTTTTTTTTTTTTTTTTTT-FAM	
	C specific	TTTTTTTTTTTTTTTTTTTTTTTTTTTCCAAAGGTCATCAACTCCCAAACG	102 bp
	T specific	TTTTTTTTTTTTTTTTTTTTTTTTTTTTTCCAAAGGTCATCAACTCCCAAACA	104 bp
rs2293347	For LDR	P-TCCAACTTCTACCGTGCCCTGATGGTTTTTTTTTTTTTTTTTTTTTTTTTTTTTTTTTT-FAM	
	A specific	TTTTTTTTTTTTTTTTTTTTTTTTTTTTTTTTTAATGCATTTGCCAAGTCCTACAGAT	118 bp
	G specific	TTTTTTTTTTTTTTTTTTTTTTTTTTTTTTTTTTTAATGCATTTGCCAAGTCCTACAGAC	120 bp
rs2072454	For LDR	P-TTGCACAGGGCAGGGTTGTTGCTGATTTTTTTTTTTTTTTTTTTTTTTTTTT-FAM	
	C specific	TTTTTTTTTTTTTTTTTTTTTTTTTTTTGTCCCGCCACTGGATGCTCTCCACG	106 bp
	T specific	TTTTTTTTTTTTTTTTTTTTTTTTTTTTTTGTCCCGCCACTGGATGCTCTCCACA	108 bp
rs28384375	For LDR	P-CGCAGTGGGGGCCGTCAATGTAGTGTTTTTTTTTTTTTTTTTTTT-FAM	
	C specific	TTTTTTTTTTTTTTTTTTTTATGACTCCTGCCGGGCAGGTCTTGG	90 bp
	T specific	TTTTTTTTTTTTTTTTTTTTTTATGACTCCTGCCGGGCAGGTCTTGA	92 bp
rs1050171	For LDR	P-TGCACGGTGGAGGTGAGGCAGATGCTTTTTTTTTTTTTTTTTTTTTT-FAM	
	A specific	TTTTTTTTTTTTTTTTTTTTTTGAAGGGCATGAGCTGCGTGATGAGT	94 bp
	G specific	TTTTTTTTTTTTTTTTTTTTTTTTGAAGGGCATGAGCTGCGTGATGAGC	96 bp
rs6965469	For LDR	P-TATATTCTTCAATGTGATCTGATAGTTTTTTTTTTTTTT-FAM	
	C specific	TTTTTTTTTTTTTTTTACAACAGCCTTCATAGTACGGCTTG	82 bp
	T specific	TTTTTTTTTTTTTTTTTTACAACAGCCTTCATAGTACGGCTTA	84 bp
rs884225	For LDR	P-GCCCCTGTCTTGCTGTCATGAAATCTTTTTTTTTTTTTTTTTTTTTTTTTTTT-FAM	
	A specific	TTTTTTTTTTTTTTTTTTTTTTTTTTTTCCATTGTTTTGAAACTCAGTATGCT	106 bp
	G specific	TTTTTTTTTTTTTTTTTTTTTTTTTTTTTTCCATTGTTTTGAAACTCAGT	108 bp

		ATGCC	
rs884904	For LDR	P-CCCTGGGGGCTGCAACTTTTAGGGATTTTTTTTTTTTTTTTTTTTTTT<br>TTTTTTT-FAM	
	A specific	TTTTTTTTTTTTTTTTTTTTTTTTTTTTTTGTGCAGAGATGATAGCTCA<br>AAATCT	110 bp
	G specific	TTTTTTTTTTTTTTTTTTTTTTTTTTTTTTTTGTGCAGAGATGATAGCTC<br>AAAATCC	112 bp
rs763317	For LDR	P-CTTTGACATTCCAGGTTTCCTCATGTTTTTTTTTTTTTTTTTTTTTTTT<br>TTTTTTTT-FAM	
	A specific	TTTTTTTTTTTTTTTTTTTTTTTTTTTTTTTTAAATGCAGAATGTGTTGC<br>ACTTTAT	114 bp
	G specific	TTTTTTTTTTTTTTTTTTTTTTTTTTTTTTTTTTAAATGCAGAATGTGTT<br>GCACTTTAC	116 bp
